# Boreal and subarctic freshwaters harbour a diversity of jumbophages

**DOI:** 10.1093/ismeco/ycag239

**Published:** 2026-08-22

**Authors:** Meeri Niemi, Karyna Karneyeva, Lotta-Riina Sundberg, Hanna M Oksanen, Elina Laanto

**Affiliations:** Department of Biological and Environmental Science and Nanoscience Center, University of Jyväskylä, PO Box 35, FI-40014 Jyväskylä, Finland; Department of Biological and Environmental Science and Nanoscience Center, University of Jyväskylä, PO Box 35, FI-40014 Jyväskylä, Finland; Department of Biological and Environmental Science and Nanoscience Center, University of Jyväskylä, PO Box 35, FI-40014 Jyväskylä, Finland; Department of Molecular and Integrative Biosciences, Faculty of Biological and Environmental Sciences, University of Helsinki, PO Box 56, FI-00014 Helsinki, Finland; Department of Biological and Environmental Science and Nanoscience Center, University of Jyväskylä, PO Box 35, FI-40014 Jyväskylä, Finland

**Keywords:** bacteriophages, freshwater ecosystems, jumbophages, boreal ecosystems, viral diversity, auxiliary metabolic genes, anti-defence systems, nucleus-forming phages, virus–host interactions

## Abstract

Bacteriophages (phages) are major drivers of microbial evolution and ecology, yet their diversity and functional roles remain poorly characterized in many natural environments, such as in freshwater systems. In boreal and subarctic freshwater habitats, where bacteria are typically slow-growing and nutrient-limited, phages are predicted to have a critical role in host regulation and horizontal gene exchange. However, only a few isolates have been obtained from such environments, leaving the genetic and functional diversity of these phages largely unexplored. Here, we present a collection of 40 bacteriophages isolated from boreal lakes and rivers using a set of diverse freshwater bacterial hosts. Despite using conventional isolation methods, eight of the isolates possess genomes larger than 200 kilobases and are classified as jumbophages. All jumbophages exhibited myovirus morphology and comparatively slow infection dynamics. These jumbophages include the first known representatives infecting members of *Janthinobacterium* and *Herbaspirillum*. Comparative genomic and phylogenetic analyses show that nearly all genomes are distinct from previously described phages, indicating substantial novelty. Diverse auxiliary metabolic and anti-defence systems were identified, including putative NAD^+^ salvage and acyl carrier protein modules, along with predicted Anti-Thoeris and Anti-CBASS elements. The *Pseudomonas-*infecting jumbophage Ahti encoded homologues of all 21 core genes that define the nucleus-forming family *Chimalliviridae.* Additionally, Ahti displayed compartmentalization of DNA during infection, establishing it as the first freshwater nucleus-forming phage. These findings expand our understanding of the ecological, genomic, and functional diversity of phages in boreal environments and highlight the role of freshwater ecosystems as significant reservoirs of novel viral lineages.

## Graphical Abstract

**Graphical Abstract:**
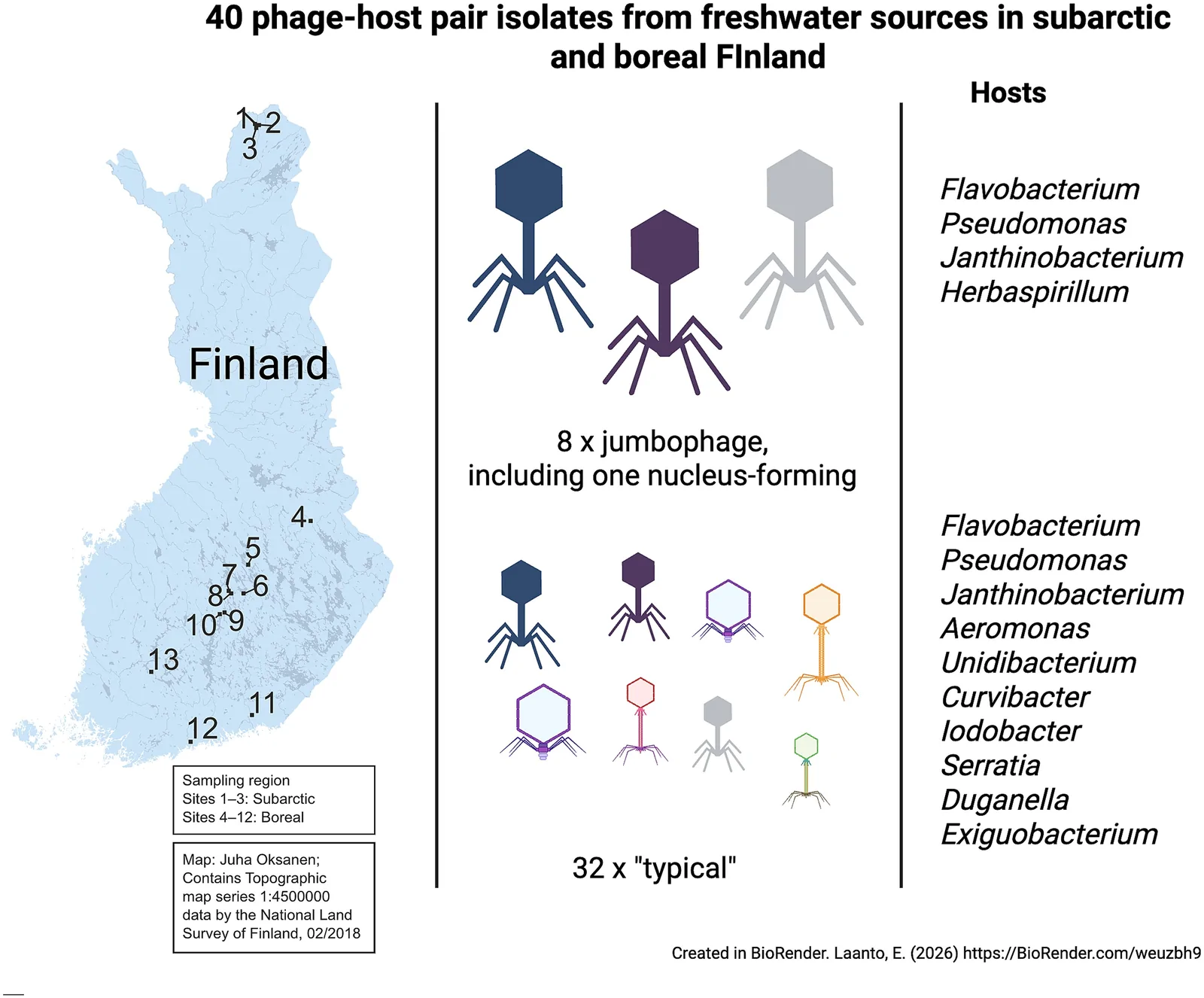

##  Introduction

The boreal biome, the world’s largest terrestrial biome, contains extensive networks of lakes, rivers, and wetlands that support diverse microbial communities adapted to cold, nutrient-poor, and humic-rich conditions [1, 2]. These ecosystems are highly vulnerable to rapid environmental change, including warming-driven shifts in water temperature, reduced ice cover duration, altered mixing regimes, and increasing terrestrial organic matter inputs that drive freshwater browning. Together, these changes can reshape habitat structure, nutrient availability, and microbial community composition in northern freshwaters [3, 4]. The functioning of these ecosystems is strongly influenced by bacteriophages (phages), which regulate their bacterial host populations, mediate horizontal gene transfer, and reprogram host metabolism [5]. In boreal freshwaters, where microbes are typically slow-growing and highly specialized [6], phages represent a major genetic reservoir. Viruses play central roles in freshwater microbial food webs by lysing host cells and redirecting cellular carbon and nutrients into dissolved organic matter through the viral shunt [7, 8]. This process can retain nutrients within microbial communities and alter the transfer of organic matter to higher trophic levels. In nutrient-poor northern freshwaters, phage-mediated host mortality, horizontal gene transfer, and auxiliary metabolic genes may therefore influence bacterial community composition, nutrient transformation, and ecosystem functioning.

Large phages, in particular, are predicted to encode many proteins with no detectable homologues in current databases or without experimentally characterized functions, including genes potentially involved in host takeover, nucleotide metabolism, translation, and anti-defence processes [9]. Despite this, environmental phages remain poorly characterized, with most current insights derived from metagenomic surveys [10]. While metagenomic approaches reveal extensive viral diversity, they are limited by challenges in assembly and host assignment. Isolate-based studies are thus essential as they provide direct evidence of phage–host interactions and enable the discovery of novel virus types [11]. Isolating unique phages not only expands our understanding of viral diversity but also allows sequence-based predictions to be linked to observable infection phenotypes, clarifying the molecular basis of phage–host interactions and their evolutionary trajectories within these complex freshwater systems.

Freshwater jumbophages represent a particularly underexplored group of such isolates. Defined by unusually large genomes exceeding 200 kilobases [12], jumbophages often encode diverse and poorly characterized functional repertoires, making them key targets for isolate-based exploration. Jumbophages have been isolated from different environments, including soil [13] and aquatic systems [14], suggesting a broad ecological distribution and prevalence. These phages exhibit several distinct biological features that set them apart from smaller, more typical and well-characterized phages. For example, some jumbophages form a proteinaceous ‘phage nucleus’ during infection, a compartmentalized structure that physically separates phage DNA from host cytoplasm, protecting replication processes from bacterial defence systems [15, 16]. Nucleus-forming jumbophages can also form early infection vesicles and encode both virion-associated and non-virion RNA polymerases, while many non-nucleus-forming jumbophages also encode their own RNA polymerases, reflecting diverse transcriptional strategies among large phages [17, 18]. Many jumbophage genomes also encode predicted anti-defence elements that may counter specific bacterial defence systems, such as Thoeris, CBASS, Gabija, or NAD^+^-depleting defence pathways [19]. In addition, jumbophage genomes can harbour auxiliary metabolic genes (AMGs) that modulate host metabolism to favour phage replication [20], as well as extensive arrays of tRNA genes that may optimize translation of phage proteins and, in some cases, contribute to evasion of tRNA-targeting host defences [21, 22]. These features highlight jumbophages as complex viruses that offer valuable insight into genome size evolution and phage–host interactions.

Despite the growing recognition of jumbophages as ecologically and evolutionarily significant, studies based on isolated phage–host systems remain limited, possibly due to the technical challenges and time-intensive nature of their isolation and characterization. Much of our current knowledge is based on metagenomic data [23], leaving substantial gaps in our understanding of jumbophage biology, diversity, and host interactions. In this study, we address this gap by characterizing a unique collection of phages isolated using multiple bacterial hosts from boreal and subarctic freshwater environments, both of which are largely unexplored in phage research. Remarkably, a significant portion of the collection (8 out of 40) were jumbophages. Five of these jumbophages infect *Flavobacterium* hosts, suggesting that members of this genus may be suitable for large-phage replication. Notably, the collection also includes the first reported jumbophages infecting members of *Herbaspirillum* and *Janthinobacterium*, two bacterial genera for which phage–host interactions remain largely unknown. Our study also reports the first nucleus-forming jumbophage isolated from freshwater. Together, these findings expand the known host range and ecological scope of jumbophages, providing valuable isolate-based data to advance the phage field.

## Materials and methods

### Environmental sampling and culture conditions

**Figure 1:**
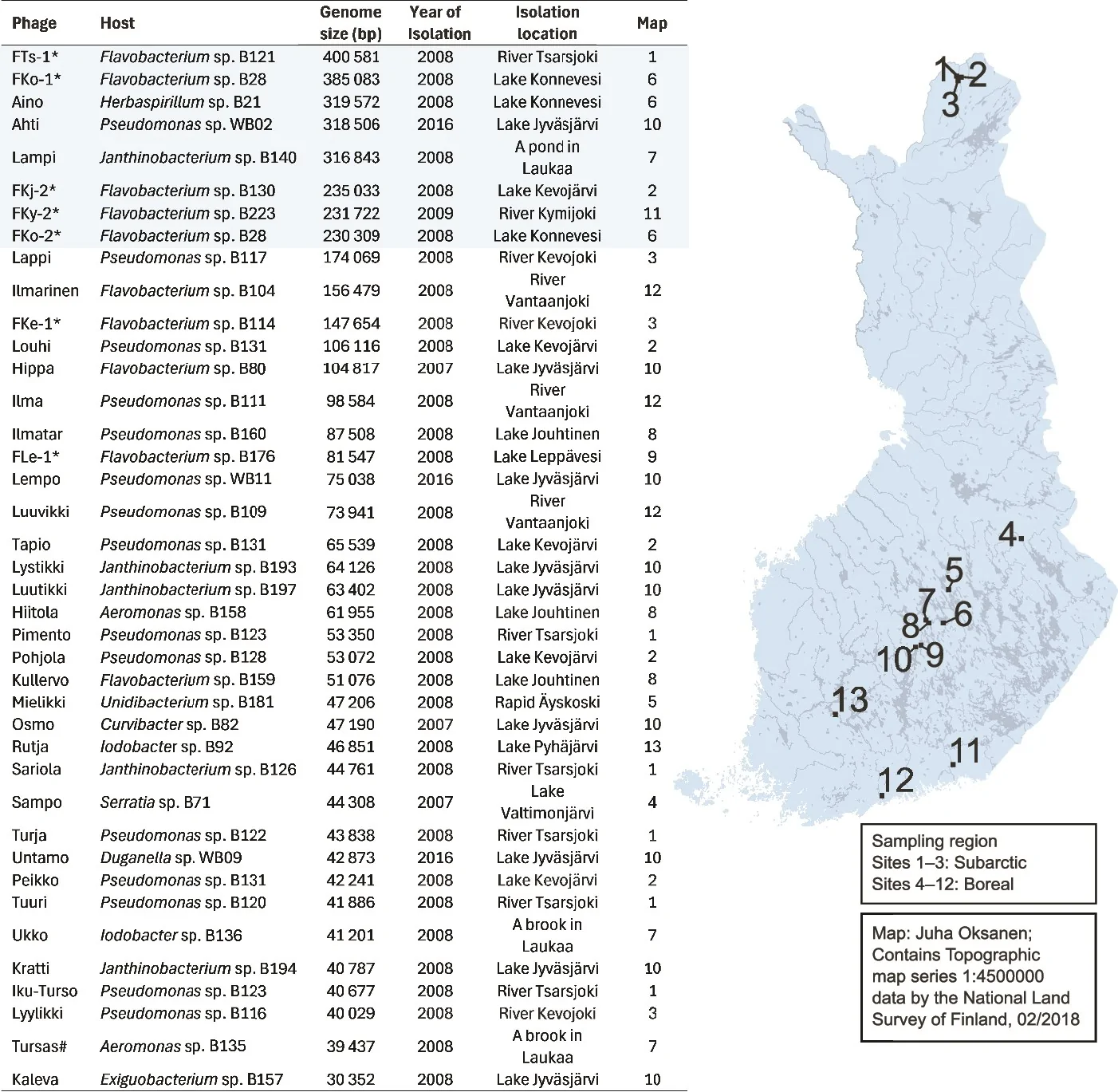
Collection of freshwater phages and their bacterial hosts arranged according to phage genome size. The phages were isolated from Finnish rivers and lakes (labelled 1–13, Table S1) in this study, except for those marked with an asterisk [48] and a hash sign [49]. Year of isolation refers to the isolation of the phage. The jumbophage isolates are highlighted in blue (the first eight entries in the table).

Freshwater samples (50 ml–2 L) were collected from surface waters of lakes and rivers across Finland (Fig. 1). These samples were used for the isolation of bacteria and their associated phages (see Phage isolation). All bacterial and phage cultures were grown in one-fifth diluted Luria–Bertani medium (1/5 LB) prepared in tap water. Solid and top-agar layers initially contained 1% and 0.5%–0.7% (w/v) agar, respectively. To improve plaque visibility, top agar was later replaced with 0.5% (w/v) low-melting agarose. Melted top agar was maintained at 47°C (agar) or 36°C (low-melt agarose) prior to plating. Unless otherwise stated, cultures were incubated aerobically at room temperature (RT; 22°C) with shaking (120 rpm).

### Bacterial host isolation and identification

Bacterial strains used as phage hosts are listed in Fig. 1 and Supplementary Table S1. New bacterial isolates were obtained by plating 100 μL of freshwater samples onto 1/5 LB agar and incubating for 2 days (RT). Single colonies were purified by forming at least three consecutive pure cultures. Host taxonomy was assigned at genus level using a 95% similarity threshold based on 16S rRNA sequence similarity to reference sequences from the NCBI database (Supplementary material, Fig. S1).

### Phage isolation and purification

Phage isolation was performed using enrichment cultures and bacterial hosts obtained from the same water source; in some cases, hosts originated from earlier sample collections at the same locations. For enrichment, water samples were filtered through 0.45 μm polyethersulfone (PES) syringe filters (Sarstedt), and LB medium was added to achieve a final 1/5 LB concentration. Overnight grown bacterial cultures were diluted tenfold into the filtrate and incubated with shaking (RT, 120 rpm) until the culture was turbid (1–2 days). Aliquots (300 μL) of the enrichment cultures were plated using the plaque assay method: 300 μL of overnight grown host culture, phage sample (diluted as necessary), and 3 ml of molten top agar were poured onto 1/5 LB agar plates and incubated for 48 h. Individual plaques were picked into 500 μL of 1/5 LB and purified through three successive rounds of single-plaque assay.

High titre phage lysates (Table S2) were prepared from semi-confluent or confluent plates by collecting top agar into 4 ml of medium per plate, shaking for 4 h (RT), centrifuging (8000 × *g*, 10 min, 4°C), and filtering (0.22 or 0.45 μm PES). Alternatively, confluent plates were overlaid with 5 ml of medium and shaken for 4–6 h (6°C, 90 rpm) before liquid collection, centrifugation (4000 × *g*, 15 min), and filtration. Jumbophage particles were further purified by PEG precipitation followed by sucrose-gradient ultracentrifugation, negatively stained, and imaged using transmission electron microscopy (Supplementary material). Lysates were stored at 4°C for short-term use or at −80°C with 20% (v/v) glycerol for long-term storage.

### Phage genome extraction, sequencing, and assembly

Phage genomic DNA was extracted from filtered lysates according to the method of Santos [24] with modifications described by Laanto and Oksanen [25]. Sequencing was performed by Novogene using the Illumina NovaSeq platform with paired-end 150 bp reads. Raw reads were uploaded to the Phagenomics phage genome analysis portal (www.phagenomics.net; accessed in February 2025) for assembly and annotation. Reads were also assembled with Velvet (version 1.2.10) in the Geneious platform (Biomatters Ltd) for comparison. When automated genome assembly by the Phagenomics portal failed, manual assemblies were conducted using the Galaxy platform [26] (Supplementary material). Final assemblies were re-uploaded to Phagenomics for downstream analyses.

### Genome annotation and comparative genomics

Genome annotation was performed via the Phagenomics portal against the PHROGs, pVOGs, COGs, and PHANNs databases (Table S3). Annotation summaries were visualized in R (v4.5.1) [27]. Jumbophages were additionally annotated using Pharokka and Phold [28] (Table S4). The closest reference genomes were identified against the INPHARED database (April 2025 release) [29] by whole-genome BLAST [30] for subsequent analysis with ViPTree (accessed in October 2025) [31], VIRIDIC (accessed in September 2025) [32], and VirClust servers (accessed in October 2025) [33].

### Functional annotation and defence systems

AMGs in the jumbophage genomes were predicted using VIBRANT (v1.2.0) [34]. Annotated AMGs were manually checked against the annotated genomes and, in the case of rare AMGs, a more detailed analysis of the surrounding genes was performed. AMG island alignments were done using DiGAlign v2.0 [35] with BLASTx. For the whole phage collection, anti-defence systems were identified with the AntiDefenseFinder mode of DefenseFinder (v2.0.2) [36], with additional searches performed for the jumbophages using DefenseFinder and PADLOC (v2.0.0) [37]. CRISPR arrays and *cas* genes were checked with CRISPRCasFinder (v1.1.2) [38]. Putative homologues of the core jumbophage genes defined by Iyer et al. [23] were identified in our genomes by searching for annotated matches. tRNA genes were extracted from all genomes using Biopython [39] in Python 3. Correlations between genome length and tRNA count were evaluated using Pearson’s and Spearman’s tests in RStudio (v2025.05.1). Plots were generated using ggplot2 (v3.5.2) [40] and dplyr (v1.1.4) [41].

### Jumbophage–host interactions

To measure the phage effect on host growth, all jumbophage hosts were grown overnight and diluted approximately 1:10 with medium to reach an OD_600_ of ~0.1. Cultures were pipetted to a Honeycomb plate (100-well plate, Oy Growth Curves Ab Ltd) with 200 μL in each well. Phage was added at three multiplicities of infection (MOIs): 0.01, 0.1, and 1 to approximately 10^7^ CFU mL^−1^ of cells in the diluted culture using a volume of 10 μL. Optical density (OD) measurements were taken with Bioscreen C (Growth Curves Ltd) at 600 nm at 15-min intervals for 25 h without shaking. All treatments were performed in five replicates, and as controls, the OD of uninfected bacteria, medium, and phage lysate were also followed. To record plaque morphology, jumbophages were spotted on their respective isolation hosts using a series of dilutions to produce distinct plaques. Following 24–48 h of incubation, plates were imaged (ChemiDoc MP, Bio-Rad).

The host range of each jumbophage was tested against all jumbophage host strains (Table S1) using a double-agar overlay spot assay. Overnight cultures (300 μL) were mixed with 3 ml soft agar and poured onto agar plates. For initial screening, 10 μL of undiluted and 1:100 diluted phage lysates were spotted onto freshly prepared bacterial lawns. Sterile medium was used as a negative control, while the original host strains were used as the positive control. Plates were incubated (RT) and examined after 24 and 48 h for clear zones of lysis or plaque formation, which were recorded as evidence of infection. Bacteria–phage pairs showing infection were then retested in triplicate, using 10-fold serial dilutions spotted onto lawns and incubated for 24 h (RT).

### Phylogenetic analyses

A curated dataset of 105 large terminase subunit sequences (Table S5), combining our 8 newly sequenced jumbophages with reference phages, was aligned with MAFFT (v7.526) [42] and analysed with IQ-TREE 2 (1000 bootstraps) [43] to infer phylogenetic relationships, with taxonomy assigned using ICTV/NCBI database and trees visualized in iTOL (v7.2.2) [44] (Supplementary material).

To assess the presence of nucleus-forming phage core genes in phage Ahti, a comparative sequence analysis was performed using the 21 core genes specific to chimallin-encoding phages as defined by Prichard et al. [45]. Protein sequences from nucleus-forming phages ΦKZ (NC_004629.1), vB_EcoM_Goslar (NC_048170.1), and vB_EamM_RAY (NC_041973.1) were used as reference sequences. The complete proteome of Ahti was queried against the core gene products using BLAST+ (BLASTp) with E-value ≤0.001, and similarity was assessed from bit scores.

### Detection of nucleus-like structures during infection

Bacterial cultures were infected, fixed, and DAPI stained (Supplementary material) to visualize DNA compartmentalization. For imaging, 2 μL of sample was placed onto 1% low-melting agarose pads and imaged by confocal microscopy. Phage nucleus formation was inferred from the appearance of centrally localized DNA focus in infected cells, in contrast to the diffuse DNA staining pattern observed in uninfected cells.

## Results

### Diverse freshwater phage–host systems recovered for genomic analysis

Across boreal and subarctic freshwater sites in Finland, the enrichment-based isolation strategy yielded 60 phage isolates, 40 of which were successfully sequenced (Fig. 1, Table S1). Sampling was conducted during the ice-free season, although a few phages were isolated from beneath the ice cover (e.g. FKo-1 and Hippa; Table S1). While a single temperature and media could lead to cultivation bias, standardized conditions were used for consistent comparison and experimental reliability. The genome-resolved phages were named using terminology from the Finnish national epic, the *Kalevala*, along with other names derived from Finnish mythology linked to aquatic environments. In total, 58 bacterial strains were isolated from freshwater samples (Table S1). Host identity was determined from partial 16S rRNA gene sequences for the 34 strains that served as hosts for phages (Fig. 1). Most isolates were gram-negative freshwater bacteria, with the only gram-positive strain belonging to *Exiguobacterium* sp., isolated from Lake Jyväsjärvi. Most isolates were assigned to *Pseudomonas* (15 strains) or *Flavobacterium* (10 strains), both common in freshwaters [46, 47]. Additional genera included *Janthinobacterium, Aeromonas, Undibacterium, Curvibacter*, and *Serratia*.

### Genome size variation highlights a subset of jumbophages

**Figure 2:**
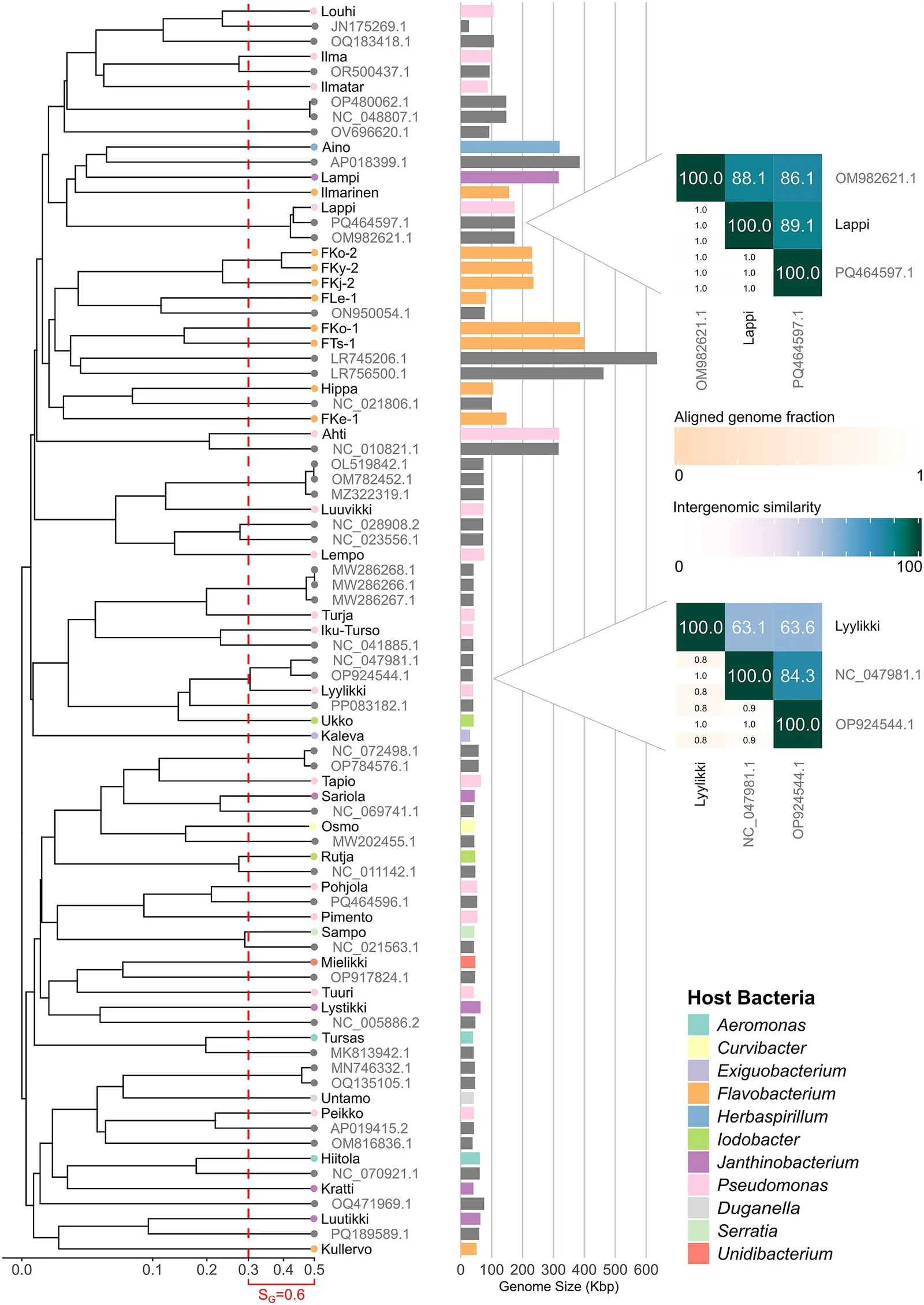
Comparison of the freshwater phages with selected reference genomes. On the left, a rectangular proteomic tree generated with the ViPTree online server is shown. Names of freshwater phages are in black, while accession numbers of reference phages retrieved from the INPHARED database are in grey. The tree was calculated from genome-wide distances based on normalized tBLASTx scores and was rooted at the midpoint. A red dashed line indicates a genomic similarity (S_G_) threshold of 0.6, used to delineate subgroups of closest relatives. On the right, a bar plot displays phage genome lengths, colour-coded by the genus of the bacterial host used for isolation (colour scheme shown in the legend, bottom right) with bars representing reference phages shown in grey. For the closest phage representatives, VIRIDIC results for Lappi and Lyytikki are provided as footnotes. Heatmaps show aligned genome fractions and intergenomic similarities for the VIRIDIC analysis.

The largest number of collected phages was isolated with *Pseudomonas* (15 out of 40), including one jumbophage. Despite *Flavobacterium-*infecting phages accounting for a smaller portion of the collection (10 out of 40), half of all *Flavobacterium* phages had genomes exceeding 200 kb. The *Flavobacterium* jumbophages formed closely related branches on the proteomic tree (Fig. 2). To further investigate their relatedness, we identified shared protein clusters (PCs) with VirClust. Protein cluster analysis (>30% identity, >50% coverage) separated the larger flavophages into two groups (Fig. S2): FKj-2, FKy-2, and FKo-2 (~200 kb) formed one clade, while FKo-1 and FTs-1 (~390 kb) grouped together on a distinct branch. VIRIDIC results mirrored this pattern, showing high relatedness only within these two clusters, with all other jumbophage pairs sharing <10% intergenomic similarity (Supplementary material Fig. S3). Larger phages also clustered separately from smaller isolates, with only sparse PC matches between them. These patterns indicate no evidence of recent genome expansion and instead support independent evolutionary lineages associated with different genome size groups.

### Freshwater phages represent novel genomic diversity

Whole-genome BLAST searches against the INPHARED database revealed no species-level relatives for any of the 40 phages. For each phage, the top-ranked genome match with the highest query coverage was selected for further analysis when available. A proteomic tree was subsequently constructed using ViPTree, including the 40 phages from this study alongside 43 reference genomes (Fig. 2). To refine the comparison for the most similar phages, they were compared using VIRIDIC. At the genus level, relatedness to previously described phages was revealed only for Lappi infecting *Pseudomonas*. It showed similarity to *Pseudomonas* phages Milchi (PQ464597.1) and Astolliot (OM982621.1), with intergenomic similarities of 89% and 88%, respectively. Both of the matching phages are currently unassigned to any genus. All other phages lacked close matches, underscoring the high diversity of obtained freshwater isolates.

Phage genomes ranged from 30 kb to 400 kb (Fig. 1, Table S1). Among the 40 genomes, 27 were below 100 kb, 5 ranged between 100 and 200 kb, and 8 exceeded 200 kb, thus qualifying as jumbophages [50]. The unexpectedly high proportion of jumbophages is notable given that all phages were obtained using conventional enrichment methods with endogenous freshwater bacteria. Five of the eight jumbophages infected *Flavobacterium* hosts, with the remaining three infecting *Pseudomonas, Janthinobacterium*, and *Herbaspirillum*. To our knowledge, these include the first reported jumbophages infecting *Janthinobacterium* and *Herbaspirillum*.

### Jumbophages display large myovirus morphology

**Figure 3:**
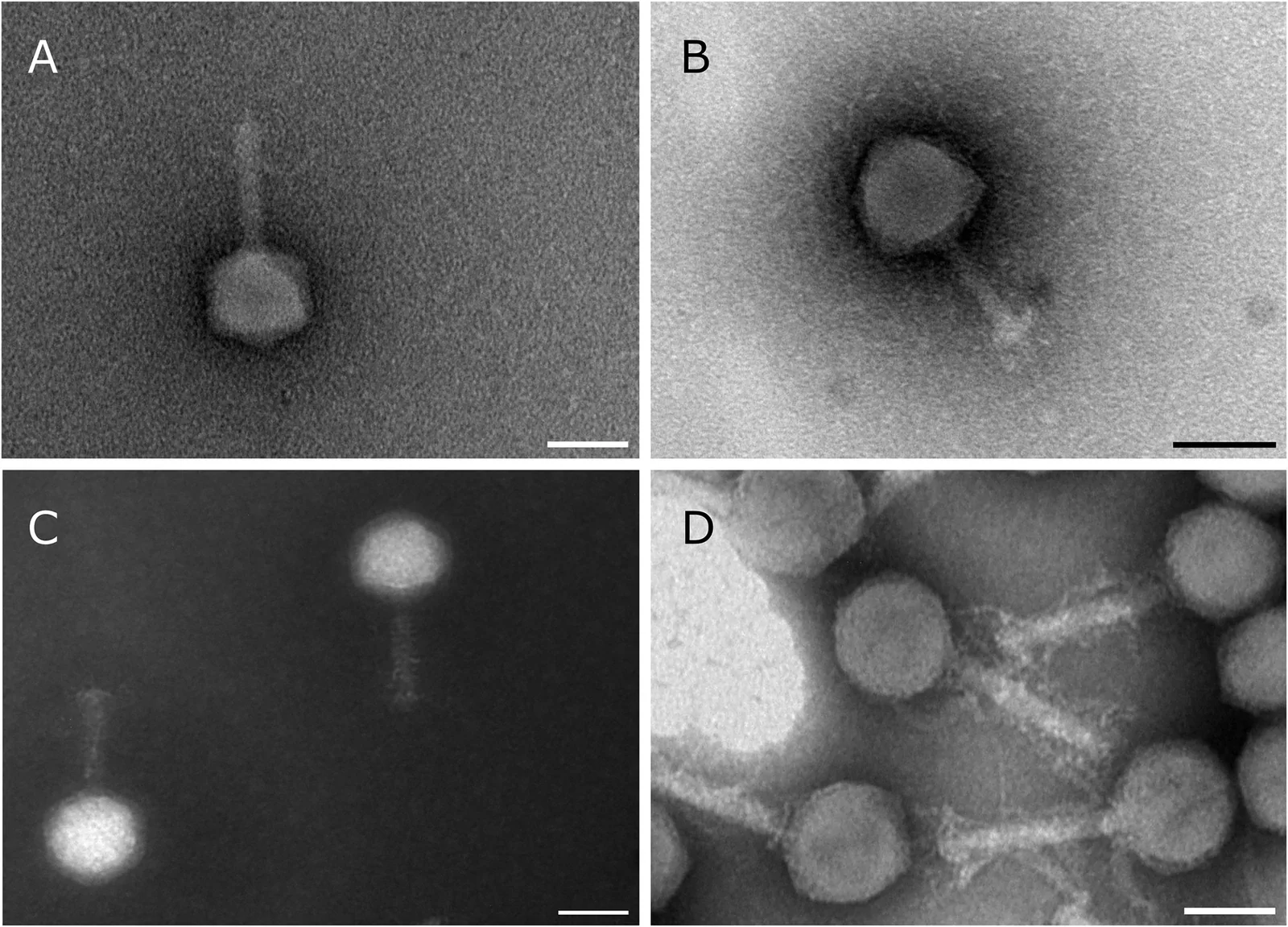
Transmission electron microscopy of negatively stained jumbophages. (A) *Flavobacterium* phage FKo-1. (B) *Herbaspirillum* phage Aino. (C) *Janthinobacterium* phage Lampi. (D) *Pseudomonas* phage Ahti. Scale bars: 100 nm.

Transmission electron microscopy revealed that all jumbophages exhibited myovirus morphology, with isometric capsids and long contractile tails (Figs 3 and S4, see also Fig. S5 for non-jumbophage isolates). FKo-2 has previously been imaged [48]. Capsid diameters (facet-to-facet) ranged from 115 to 149 nm (mean = 131 nm), while tail lengths varied from 112 to 187 nm (mean = 157 nm) (Table S6). Janthinobacterium infecting phage Lampi had the largest virion with a capsid diameter of 146 nm and a tail length of 141 nm. Overall, morphology was consistent across isolates, suggesting a shared structural architecture despite their genomic diversity (see below).

### Jumbophage–host interactions

**Figure 4:**
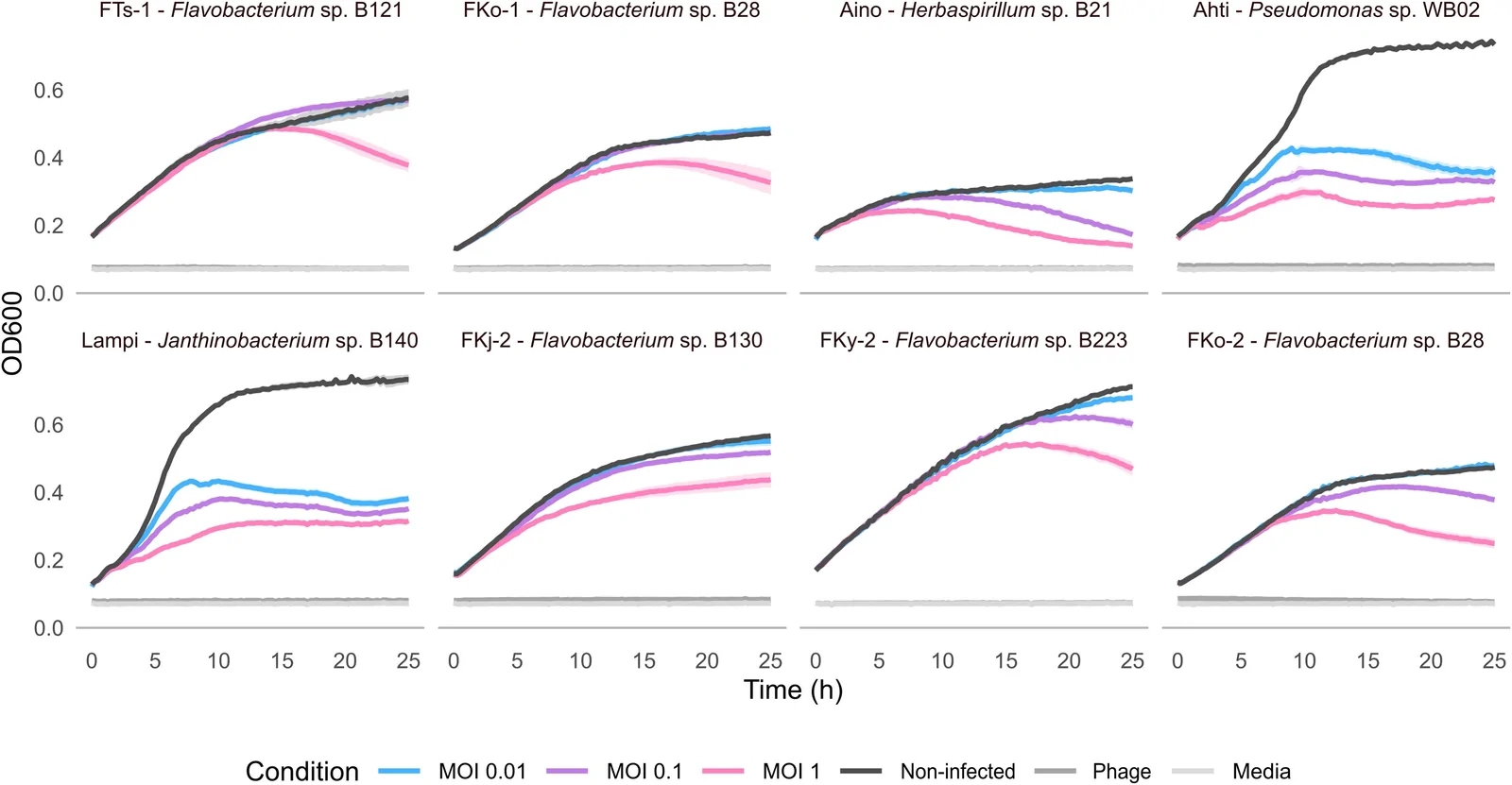
Jumbophage infections. Optical density (OD_6_₀₀) measurements of host cultures infected with each jumbophage at three MOIs (0.01, 0.1, and 1). Each host–phage panel represents an individual phage isolate, with its host strain. Curves represent the mean OD_6_₀₀ of five replicate wells, and shading indicates ±SD.

For most jumbophage–host pairs, low-MOI infections (0.01–0.1) resulted in growth curves indistinguishable from uninfected controls, suggesting limited infection efficiency at low initial phage-to-cell ratios (Fig. 4). In contrast, several phages exhibited clear inhibitory and lytic effects, such as a reduced growth rate, at MOI 1. Overall, these results indicate a strong MOI dependence in detectable culture-level effects. This pattern is consistent with the longer latent periods reported for some jumbophages [51–53].

**Figure 5:**
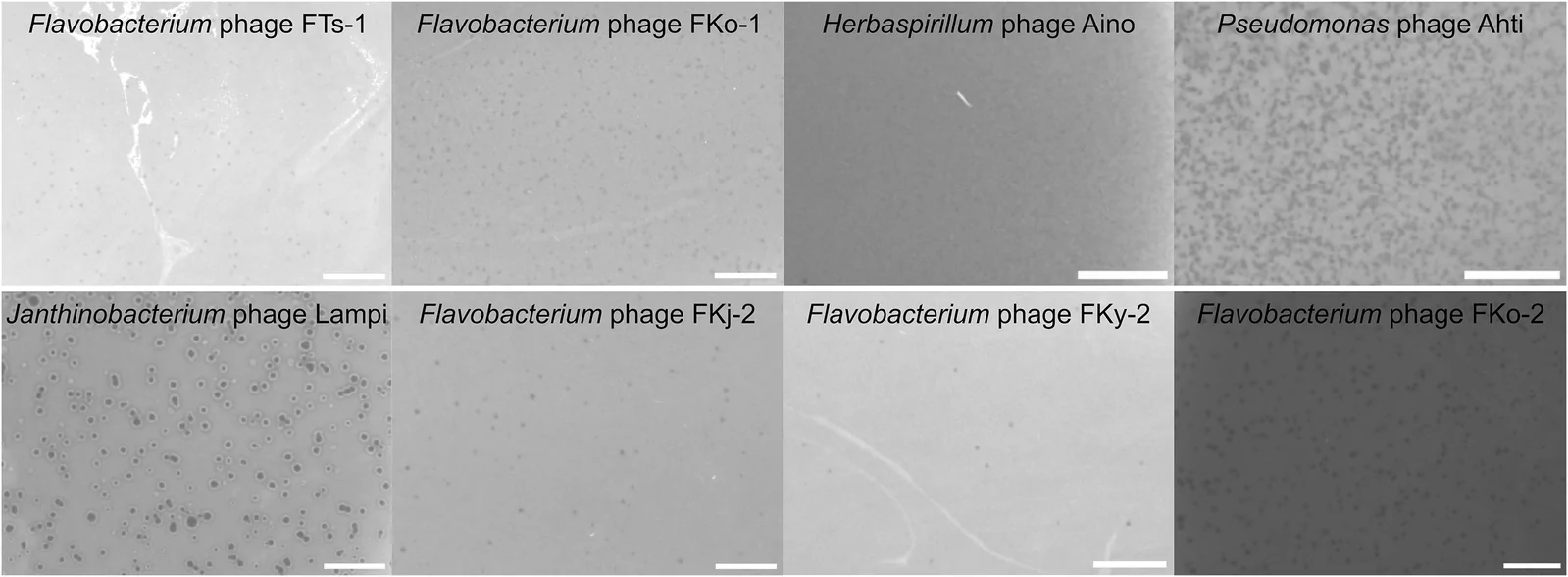
Plaque morphologies of the jumbophages on their isolation host bacteria at 24–48 h post-infection. Scale bars: 5 mm.

Host range assays conducted across all jumbophage hosts showed that all jumbophages infected only bacterial strains belonging to the same genus as their original isolation host, indicating a narrow host range (Fig. S6). In addition, when tested against 12 *Pseudomonas* sp. strains, Ahti infected 2 additional strains: *Pseudomonas* sp. B117 and *Pseudomonas* sp. B122. On their isolation hosts, the jumbophages predominantly formed small plaques (Fig. 5, Fig. S7). *Herbaspirillum* phage Aino formed particularly small, turbid plaques, consistent with low infection efficiency, incomplete lysis or poor host growth (Fig. 4), which made quantitative assessment of plaques difficult.

**Figure 6:**
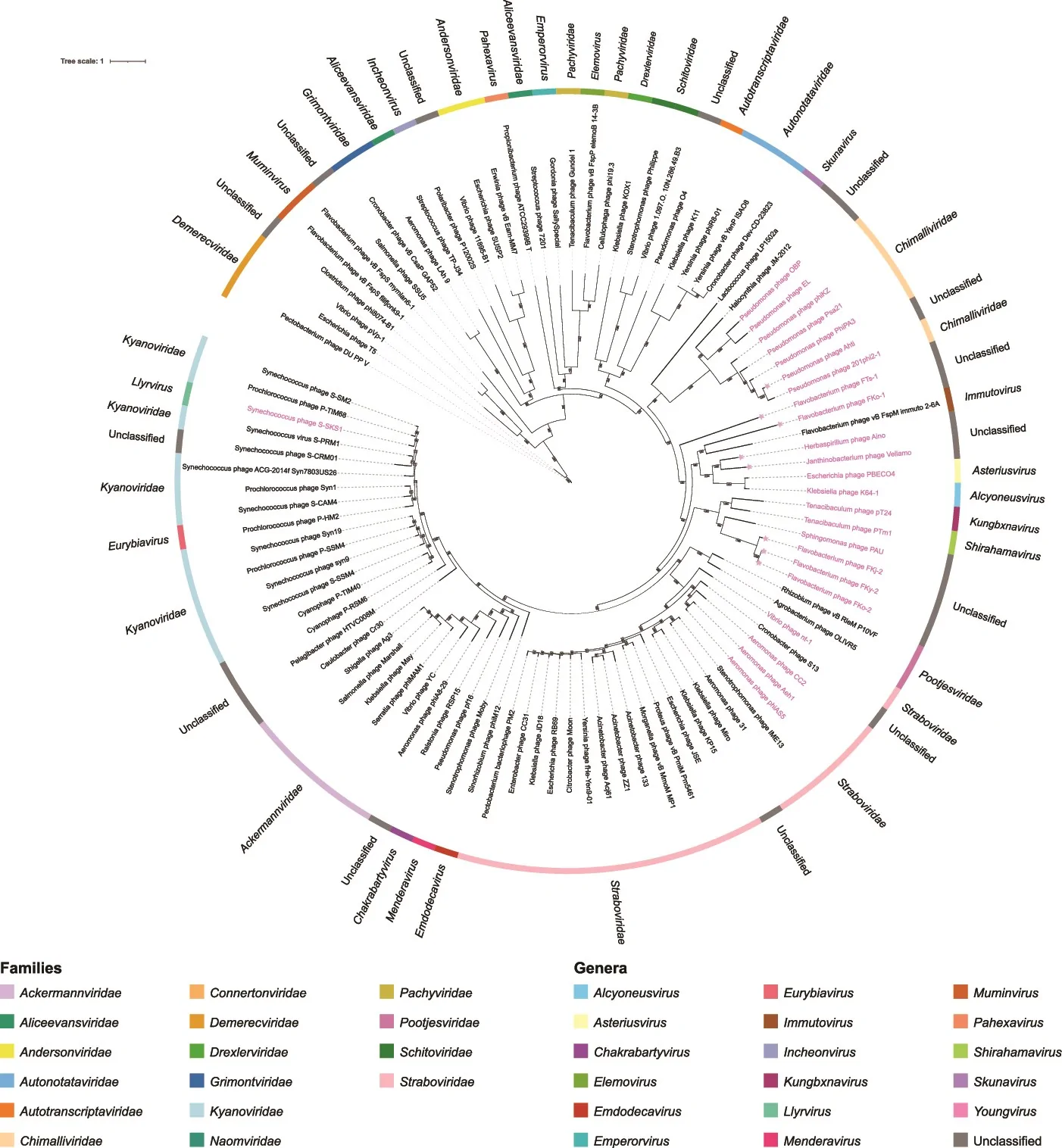
Maximum likelihood phylogenetic tree of large terminase subunit protein sequences from the eight freshwater jumbophages (stars) and 97 representative reference sequences from diverse tailed dsDNA phage lineages. Jumbophages are marked in pink. Analyses were performed using IQ-TREE with the best-fit evolutionary model selected by model testing and 1000 bootstrap replicates. Bootstrap values are indicated for each node. The tree is midpoint-rooted and visualized in iTOL v7. The outer ring is colour-coded by phage family or genus (when not assigned to a family).

Phylogenetic analysis based on the large terminase subunit positioned the newly isolated jumbophages among established dsDNA phage lineages (Fig. 6, see also Fig. S8 for major capsid protein phylogeny). The eight new jumbophages did not form a single monophyletic cluster but instead grouped separately with representatives of multiple phage lineages. The *Flavobacterium*-infecting phages FKo-2, FKy-2, and FKj-2 clustered closely together, consistent with their higher intergenomic similarity values. In contrast, *Janthinobacterium* phage Lampi and *Herbaspirillum* phage Aino were positioned in more distant branches. Bootstrap support was high (>70) for most major nodes, confirming the reliability of the observed relationships. Collectively, the tree highlights the phylogenetic diversity of the jumbophages and their distribution across multiple branches, consistent with the broader polyphyletic nature of jumbophages reported previously [23, 50].

### Functional annotation highlights diverse biological capacities

Genome annotation revealed substantial variation in functional content across the phage collection, with larger genomes containing a higher proportion of unannotated genes and more putative metabolic- and host-interaction–related functions than the smaller ones (Supplementary material Fig. S9). The *Pseudomonas* jumbophage Ahti uniquely encoded numerous head-and-packaging genes, while the largest genomes showed increased COG database matches, including a ribosomal protein in Aino. Given that larger phage genomes are known to encode more tRNAs [9, 54], we examined tRNA gene content within our collection. Phage-encoded tRNAs may help compensate for differences between viral and host codon usage and support efficient translation of phage proteins during infection [54]. Predicted tRNA counts ranged from 0 to 62 and tended to increase with genome size, yet several of the largest jumbophages encoded only a few tRNAs or none at all (Supplementary material, Fig. S10).

Across the collection, we identified genes representing all major functional categories implicated in the control and modification of host metabolism (Tables S3 and S4). A broad array of genes associated with transcription and translation was present in all genomes. The phages Luuvikki and Lempo each encoded a putative virion RNA polymerase, while the two largest jumbophages, FKo-1 and FTs-1, carried two distinct RNA polymerase σ-factor variants per genome. In the *Flavobacterium* phage Hippa, a large, predicted protein (2175 residues; gene V2_00052) contained the conserved DFDID motif, characteristic of distant RNA polymerases previously described in crAss-like phages [55].

Several large genomes also encoded tRNA synthetases and proteins involved in tRNA modification or ligation, whereas a translation initiation factor was predicted exclusively in the largest FTs-1 genome. Genes related to host cell lysis were detected in all phages except Tursas and Kullervo. In contrast, genes potentially associated with temperate replication were restricted to smaller phages. Notably, an ‘excisionase and transcriptional regulator’ was predicted only for the smallest phage in the collection, Kaleva.

### Metabolic potential of the jumbophage isolates: candidates for AMGs

**Figure 7:**
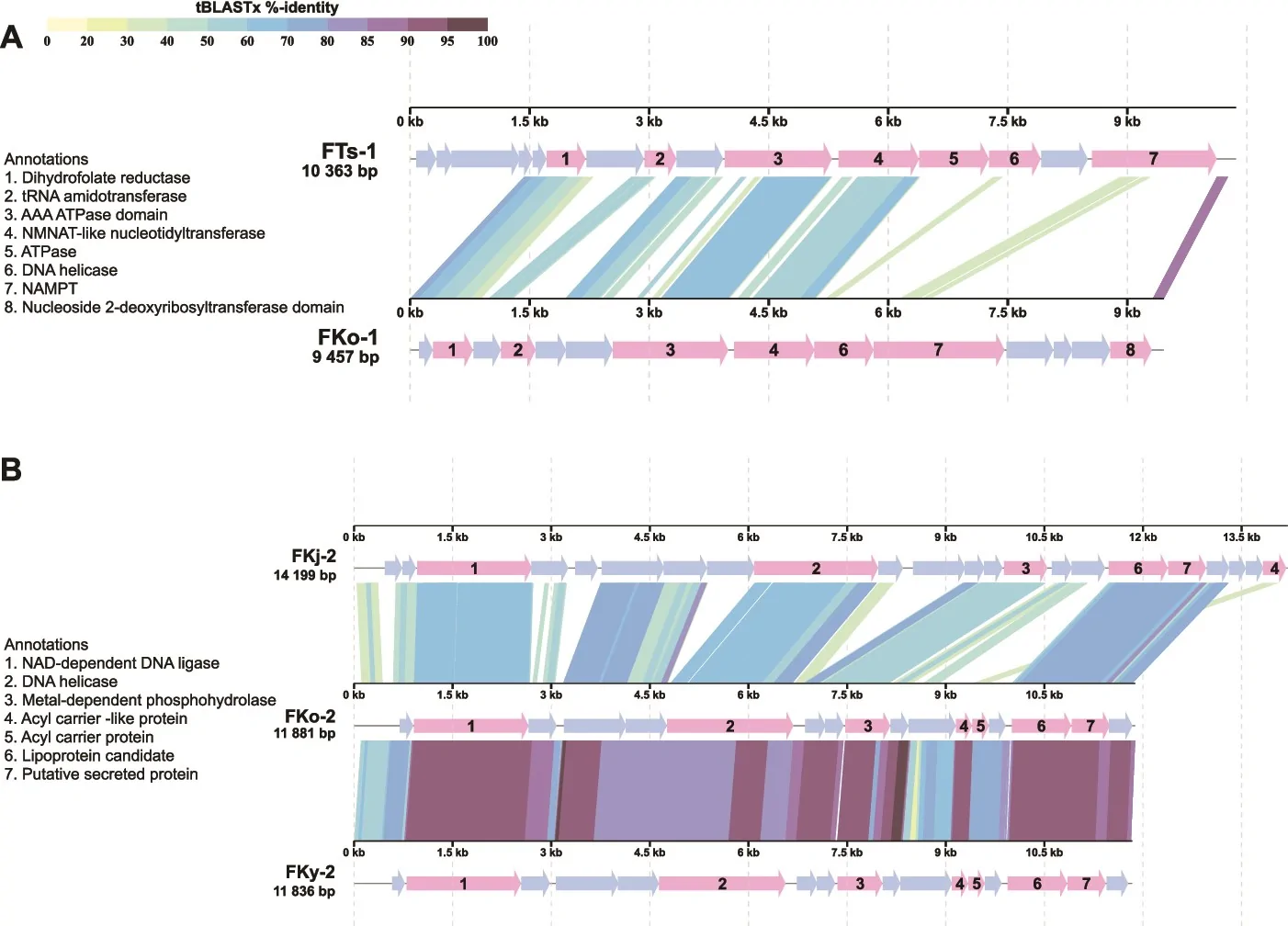
AMG islands in freshwater jumbophages. Alignments highlighting the two major putative AMG modules identified in the jumbophage genomes. (A) NAD–salvage module in jumbophages FTs-1 and FKo-1. (B) Host-interface island in jumbophages FKj-2, FKo-2, and FKy-2. Coloured ribbons indicate homologous proteins (tBLASTx percent identity). Genes with functional annotations are highlighted in pink.

We identified putative AMGs from jumbophages (Table S7). A phage-encoded enzyme, a nicotinamide phosphoribosyltransferase (NAMPT), not previously detected in jumbophages, was present in the largest phages Ahti, Aino, FTs-1, and FKo-1. This enzyme, part of the NAD^+^ salvage pathway [56], has previously been reported in Vibrio phage KVP40 [57]. In the *Flavobacterium* jumbophages FTs-1 and FKo-1, the NAMPT gene occurs within a ~10 kb long conserved gene cluster in which other genes related to NAD-salvage were also detected (Fig. 7, Table S8) suggesting the potential for NAD metabolism-related functions. Here, we refer to these clusters as phage NAD-salvage islands. For both *Flavobacterium* jumbophages, the islands include a gene encoding a protein with a likely bifunctional architecture: an N-terminal nucleotidyltransferase (NMNAT-like) domain and a C-terminal Nudix hydrolase domain. This gene is located near the NAMPT gene, suggesting possible co-regulation.

Another distinct putative AMG module was identified in the *Flavobacterium* phages FKo-2, FKj-2, and FKy-2 (Fig. 7, Table S8) that we named Host-interface island. For example, in FKo-2 we identified a 17-gene island with secreted and lipoprotein candidates, duplicated acyl-carrier proteins, and a metal-dependent phosphohydrolase. This gene composition suggests a potential role in host interaction, possibly related to surface processes such as adsorption or envelope modification. In FKj-2, the annotated acyl carrier proteins (ACPs) differ at the nucleotide level from those of the other two phages, but the overall proximity of genes is retained. While phage-encoded ACPs and secreted effectors have been reported in other jumbophages [58], the recurrent and syntenic occurrence of this multi-gene module across different genomes could suggest it represents a conserved feature among freshwater flavobacterial phages. Thus, we suggest this to be a putative phage-encoded module raising the possibility of phage-mediated modulation of host membrane components.

### Anti-defence systems are prevalent in jumbophages

Genomic analyses revealed multiple putative anti-defence systems, with detection varying slightly depending on the annotation method used (Phagenomics versus Pharokka + Phold). Among the non-jumbophages, 7 out of 32 genomes contained at least 1 predicted anti-defence element. In contrast, five of the eight jumbophages contained at least one predicted anti-defence system, with several encoding two. Within the jumbophages, putative Anti-Thoeris elements were the most frequently identified and included tad1 in Ahti and atd1 in FKo-1, FTs-1, and Lampi by at least one annotation-tool combination. Putative Anti-CBASS acb1 elements were detected in FKo-1 and Aino, while an Anti-Gabija gad1 element was detected only in Aino. Additionally, Ahti encoded a putative NAD^+^ reconstitution pathway 1 (NARP1), listed under the NADP category in AntiDefenseFinder, which represents a predicted phage anti-defence strategy against NAD^+^-depleting bacterial defence systems [19, 59] (Table S9). Separately, PADLOC detected PDC-S10 phage defence candidate loci in FKo-1 and FTs-1 with both annotation pipelines.

No CRISPR arrays or *cas* genes were found in any of the jumbophage genomes. Comparative searches for the broadly conserved, but not universally present, jumbophage protein families analysed by Iyer et al. [23] showed that most were absent from the jumbophage genomes described here, with only a small subset of replication- and repair-associated homologues detected (Fig. S11). These findings suggest that the freshwater jumbophages possess distinct functional repertoires compared with previously characterized marine and *Pseudomonas* jumbophages [23].

### Pseudomonas jumbophage Ahti is a freshwater nucleus-forming phage

**Figure 8:**
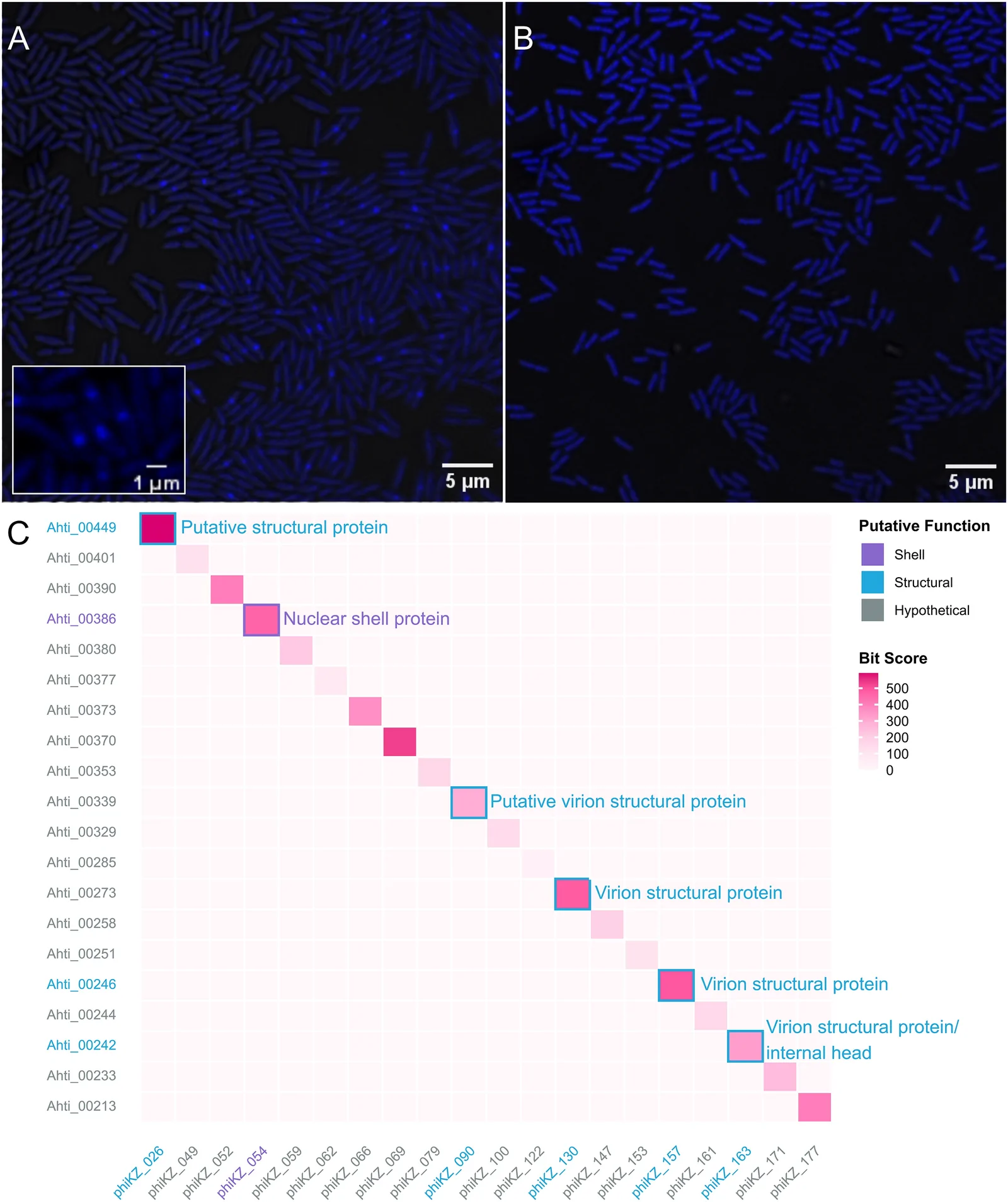
Nucleus-like compartment formation and presence of nucleus-forming core gene products in phage Ahti. (A) Confocal microscopy of *Pseudomonas* sp. WB02 infected with phage Ahti at 60 min post-infection, shown as an overlay of brightfield and DAPI channels. DAPI-stained DNA shows distinct intracellular localization, consistent with formation of a nucleus-like compartment. The inset shows a zoomed-in view of infected cells with focused DAPI signal. (B) Uninfected control cells shown as an overlay of brightfield and DAPI channels, displaying diffuse DAPI staining. Scale bars: 5 μm in the main images and 1 μm in the inset. (C) Heatmap of BLASTp bit scores (0–500) comparing the Ahti proteome to the 21 core gene products defined as exclusive to nucleus-forming phages [45]. ΦKZ was used as the reference genome for these core genes due to its genetic similarity to Ahti. Because ΦKZ lacks 1 core gene and contains 3 paralogous copies of another (for which only the highest-scoring match is shown), the heatmap displays 20 unique core-gene matches. Gene products are colour-coded by predicted functional class, and text labels indicate putative functions of the corresponding ΦKZ genes where specific functional annotations are available. Unlabelled grey entries correspond to core genes annotated as hypothetical proteins in Prichard et al. [45].

During infection of *Pseudomonas* sp. WB02 by phage Ahti, confocal microscopy revealed distinct localization of DAPI-stained DNA within host cells, in contrast to the diffuse staining pattern observed in the uninfected control (Fig. 8A and B). This indicates compartmentalization of phage DNA during infection, consistent with the formation of a nucleus-like structure [16]. No comparable localization was detected for any other freshwater jumbophage infection (Fig. S12). Genomic analysis revealed that Ahti encodes homologues of predicted tubulin and chimallin proteins, which are associated with known nucleus-forming phages [45, 60]. Furthermore, Ahti contained homologues for all 21 core genes defined as exclusive to nucleus-forming phages of the family *Chimalliviridae* [45], most of which are annotated as hypothetical proteins (Figs 8C and S13, Table S10). Additional targeted searches detected a strong candidate homologue of PicA/Imp1, a protein-import-associated factor described in nucleus-forming phages, as well as multiple RNA polymerase-associated marker proteins (Table S11) [61]. Within these core-gene and marker-protein comparisons, the strongest Ahti matches were to ΦKZ, with weaker similarity to Goslar and RAY, consistent with ΦKZ being the closest of the three reference phages to Ahti in whole-proteome comparison (Table S12). Terminase-based phylogeny further placed Ahti within *Chimalliviridae* (Fig. 6). Together, these findings indicate that Ahti represents a new member of the family *Chimalliviridae* and is, to our knowledge, the first nucleus-forming freshwater jumbophage.

## Discussion

Metagenomic surveys consistently highlight the immense viral diversity of aquatic systems [62–64]. However, isolate-based studies remain essential to validate phage–host interactions and test predicted functions experimentally. While 80% of metagenomics datasets are derived from environmental samples (IMG/VR database), and marine environments are recognized as hotspots of phage diversity [65], phage banks are dominated by soil- and clinic-derived isolates. Systematic collections of environmental isolates remain scarce. By isolating diverse endogenous phage–host pairs from freshwaters, we established a unique collection that reflects ecologically relevant interactions. Our results show that boreal and subarctic freshwater environments serve as an underexplored reservoir of unique non-model systems.

In total, 40 unique phages were isolated infecting bacterial hosts from 11 phylogenetically diverse genera. A high proportion of jumbophages was surprising considering the use of conventional enrichment-based isolation methods and endogenous host bacteria. These jumbophages span diverse evolutionary lineages, including the first known jumbophage isolates infecting *Herbaspirillum* and *Janthinobacterium*. In addition, we describe one *Pseudomonas* jumbophage and five *Flavobacterium* jumbophages. Jumbophages showed no evidence of a single origin. Instead, they formed distinct lineages, including two divergent *Flavobacterium-*infecting groups with genome sizes of ~235 kb and 390 kb. These patterns reflect long-term evolutionary separation and high genomic diversity.

Large genome size is unusual among bacteriophages, which rely on host resources for replication. In jumbophages, expanded genome size may support broader functional capacity and reduce reliance on host metabolic processes, potentially enabling more complex infection strategies [50]. Jumbophages can fine-tune host resource utilization through AMGs. We identified two putative AMG islands in *Flavobacterium*-infecting jumbophages, one centred on NAD–salvage and another on putatively modifying the host cell surface or envelope. The NAD–salvage island can aid in viral replication by boosting NAD^+^ availability. In turn, the Host-interface island putatively supports host biosynthetic activity during infection. Their presence across jumbophages suggests selective advantages in nutrient-limited and variable freshwater environments. These islands may represent transferable functional modules shaping phage–host interactions in lakes and rivers. While marine and *Pseudomonas* jumbophages have been shown to encode certain core functions and adaptive features [23], the freshwater phages stand out, since they lack homologues to most of these genes. These findings emphasize that jumbophages do not share a standard functional repertoire but instead employ diverse genomic strategies to thrive in different ecological contexts.

A larger genome may also confer jumbophages with more efficient strategies for host takeover. In our jumbophages, we detected transcription- and translation-related proteins and a diverse repertoire of predicted tRNAs, which may also increase replication efficiency [66]. Moreover, recent bioinformatic evidence indicates that some phages even encode ribosomal proteins [67], potentially representing a novel strategy for manipulating host translation. Their functionality, however, remains experimentally unvalidated [67]. Notably, within our collection, *Herbaspirillum* phage Aino was predicted to encode a ribosomal protein L7/L12 homologue, highlighting Aino’s potential as an experimental model for assessing whether such genomic predictions reflect real biological function.

Host defence systems can be detrimental to phage propagation [68]. Thus, another way for jumbophages to benefit from their increased genomic capacity is to ensure a successful infection despite these defences. A variety of anti-defence systems have been described for phages [69], and in our collection, a proportionally higher number of jumbophages had predicted anti-defence elements compared to the smaller phages. Given the high proportion of unannotated genes among large genomes, the likelihood to discover yet-uncharacterized systems remains high. Jumbophage genomes can also encode more complex infection strategies, such as the formation of a protective phage nucleus. In nucleus-forming phages, this compartment can protect phage DNA from tested DNA-targeting defence systems, including restriction–modification and CRISPR–Cas systems. This feature distinguishes members of the family *Chimalliviridae* [45]. In our study, *Pseudomonas* phage Ahti exhibited this strategy, as confirmed by confocal microscopy. Genomic analysis further supported this observation: Ahti harbours a tubulin gene as well as homologues to all 21 core genes defined for *Chimalliviridae* [45]. Furthermore, Ahti clusters with known members of this family in terminase-based phylogenetic analysis*.* We therefore propose Ahti as a new species of the family *Chimalliviridae*, representing, to our knowledge, the first nucleus-forming jumbophage isolated from freshwater.

Overall, our study underscores boreal and subarctic freshwaters as rich reservoirs of non-model bacterium–phage systems, revealing novel jumbophage lineages, new host associations, and diverse biological strategies. While host identity is likely a major determinant of phage diversity, the boreal freshwater environments sampled here may also provide ecological conditions that favour cold-adapted bacterial hosts and the jumbophages associated with them. The observed diversity, therefore, likely reflects both the availability of suitable hosts and the selective pressures imposed by boreal freshwater habitats. Described isolates provide cultured representatives of the viral ‘dark matter’ frequently observed in metagenomic surveys, transforming sequence-based predictions into tangible biological entities. This jumbophage collection offers valuable experimental model systems to challenge the traditional boundary between cellular and non-cellular life. By expanding the taxonomic spectrum of known phages, these cultured isolates offer a foundation for investigating how jumbophages shape freshwater microbiomes and drive ecosystem dynamics.

## Supplementary Material

Supplementary_material_ycag239

## Data Availability

All sequences generated in this study have been submitted to NCBI, and accession numbers are listed in Table S1. Phage genome accessions are PZ831762–PZ831801, and bacterial partial 16S rRNA accessions are PZ822963–PZ822998.
