# Supplementary material for: Boreal and subarctic freshwaters harbour a diversity of jumbophages
Source: ISME Commun. 2026 Aug 22;6(1):ycag239. doi: 10.1093/ismeco/ycag239 (PMC13573597; doi:10.1093/ismeco/ycag239)
Supplement: Supplementary file 1 [file supplementary_material_ycag239.zip › figS2 (1).pdf]

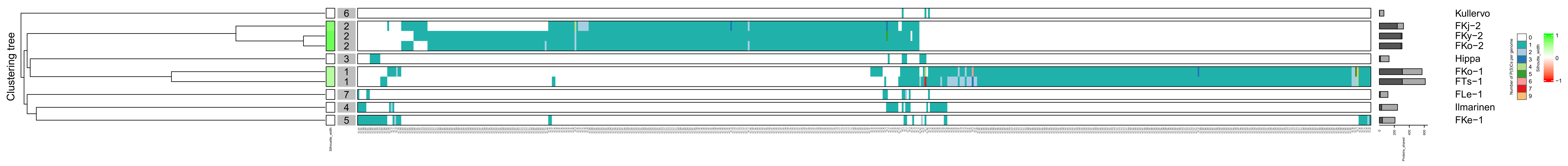
Clustering tree
6
2
2
2
3
1
1
7
4
5
Kullervo
FKj-2
FKy-2
FKo-2
Hippa
FKo-1
FTs-1
FLe-1
Ilmarinen
FKe-1
