# Supplementary material for: Boreal and subarctic freshwaters harbour a diversity of jumbophages
Source: ISME Commun. 2026 Aug 22;6(1):ycag239. doi: 10.1093/ismeco/ycag239 (PMC13573597; doi:10.1093/ismeco/ycag239)
Supplement: Supplementary file 1 [file supplementary_material_ycag239.zip › figS8.pdf]

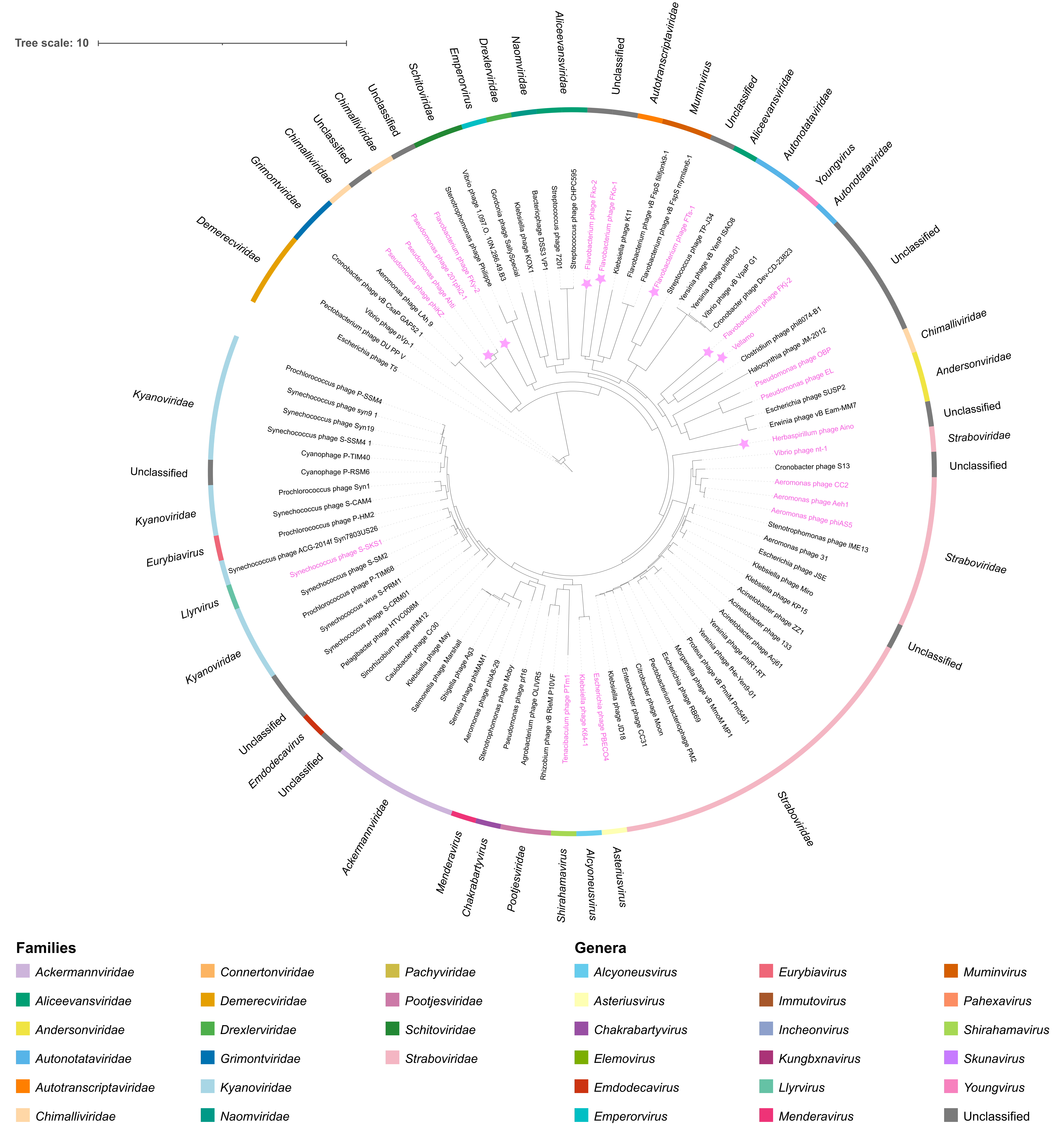

Tree scale: 10
Demerecviridae
Grimontviridae
Chimalliviridae
Unclassified
Chimalliviridae
Unclassified
Schitoviridae
Emperorvirus
Drexlerviridae
Naomviridae
Aliceevansviridae
Unclassified
Autotranscriptaviridae
Muminvirus
Unclassified
Aliceevansviridae
Autonotataviridae
Youngvirus
Autonotataviridae
Unclassified
Chimalliviridae
Andersonviridae
Unclassified
Straboviridae
Unclassified
Straboviridae
Unclassified
Straboviridae
Asteriusvirus
Alcyoneusvirus
Shirahamavirus
Pootjesviridae
Chakrabartyvirus
Menderavirus
Ackermannviridae
Unclassified
Emdodecavirus
Unclassified
Kyanoviridae
Llyrvirus
Eurybiavirus
Kyanoviridae
Unclassified
Kyanoviridae
Escherichia phage T5
Pectobacterium phage DU PP V
Vibrio phage pVp-1
Cronobacter phage vB CsaP GAP52 1
Aeromonas phage LAh 9
Pseudomonas phage phiKZ
Pseudomonas phage Ahti
Pseudomonas phage 201phi2-1
Flavobacterium phage FKy-2
Stenotrophomonas phage Philippe
Vibrio phage 1.097 O. 10N.286.49 B3
Gordonia phage SallySpecial
Klebsiella phage KOX1
Bacteriophage DSS3 VP1
Streptococcus phage 7201
Streptococcus phage CHPC595
Flavobacterium phage Fko-2
Flavobacterium phage FKo-1
Klebsiella phage K11
Flavobacterium phage vB FspS filifjonk9-1
Flavobacterium phage vB FspS mymlan6-1
Flavobacterium phage FIs-1
Streptococcus phage TP-J34
Yersinia phage vB YenP ISAO8
Yersinia phage phiR8-01
Vibrio phage vB VpaP G1
Cronobacter phage Dev-CD-23823
Flavobacterium phage FKj-2
Vellamo
Clostridium phage phi8074-B1
Halocynthia phage JM-2012
Pseudomonas phage OBP
Pseudomonas phage EL
Escherichia phage SUSP2
Erwinia phage vB Eam-MM7
Herbaspirillum phage Aino
Vibrio phage nt-1
Cronobacter phage S13
Aeromonas phage CC2
Aeromonas phage Aeh1
Aeromonas phage phiAS5
Stenotrophomonas phage IME13
Aeromonas phage 31
Escherichia phage JSE
Klebsiella phage Miro
Klebsiella phage KP15
Acinetobacter phage ZZ1
Acinetobacter phage 133
Acinetobacter phage Acj61
Yersinia phage phiR1-RT
Yersinia phage fHe-Yen9-01
Proteus phage vB PmiM Pm5461
Morganella phage vB MmoM MP1
Escherichia phage RB69
Pectobacterium bacteriophage PM2
Citrobacter phage Moon
Enterobacter phage CC31
Klebsiella phage JD18
Escherichia phage PBECO4
Klebsiella phage K64-1
Tenacibaculum phage PTm1
Rhizobium phage vB RleM P10VF
Agrobacterium phage OLIVR5
Pseudomonas phage pf16
Stenotrophomonas phage Moby
Aeromonas phage phiA8-29
Serratia phage phiMAM1
Shigella phage Ag3
Salmonella phage Marshall
Klebsiella phage May
Caulobacter phage Cr30
Sinorhizobium phage phiM12
Pelagibacter phage HTVC008M
Synechococcus phage S-CRM01
Synechococcus virus S-PRM1
Prochlorococcus phage P-TIM68
Synechococcus phage S-SM2
Synechococcus phage S-SKS1
Synechococcus phage ACG-2014f Syn7803US26
Prochlorococcus phage P-HM2
Synechococcus phage S-CAM4
Prochlorococcus phage Syn1
Cyanophage P-RSM6
Cyanophage P-TIM40
Synechococcus phage S-SSM4 1
Synechococcus phage Syn19
Synechococcus phage syn9 1
Prochlorococcus phage P-SSM4
Families
Ackermannviridae
Aliceevansviridae
Andersonviridae
Autonotataviridae
Autotranscriptaviridae
Chimalliviridae
Connertonviridae
Demerecviridae
Drexlerviridae
Grimontviridae
Kyanoviridae
Naomviridae
Pachyviridae
Pootjesviridae
Schitoviridae
Straboviridae
Genera
Alcyoneusvirus
Asteriusvirus
Chakrabartyvirus
Elemovirus
Emdodecavirus
Emperorvirus
Eurybiavirus
Immutovirus
Incheonvirus
Kungbxnavirus
Llyrvirus
Menderavirus
Muminvirus
Pahexavirus
Shirahamavirus
Skunavirus
Youngvirus
Unclassified
