# Supplementary material for: Boreal and subarctic freshwaters harbour a diversity of jumbophages
Source: ISME Commun. 2026 Aug 22;6(1):ycag239. doi: 10.1093/ismeco/ycag239 (PMC13573597; doi:10.1093/ismeco/ycag239)
Supplement: Supplementary file 1 [file supplementary_material_ycag239.zip › figS11.pdf]

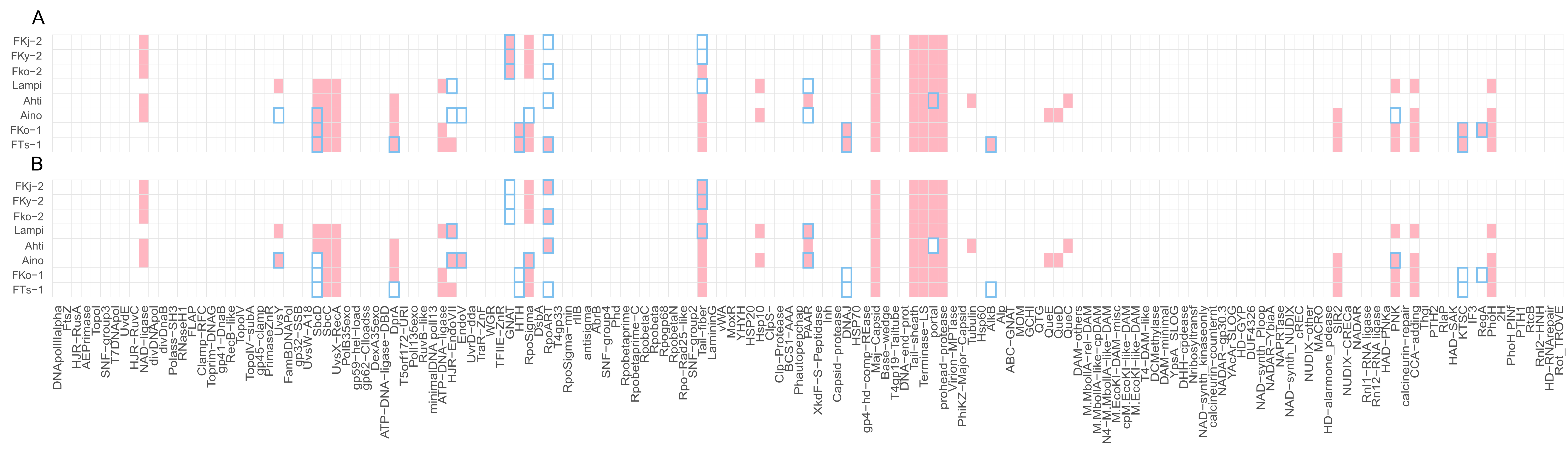
A
FKj−2
FKy−2
Fko−2
Lampi
Ahti
Aino
FKo−1
FTs−1
B
FKj−2
FKy−2
Fko−2
Lampi
Ahti
Aino
FKo−1
FTs−1
DNApolIIIalpha
FtsZ
HJR−RusA
AEPrimase
TopoI
SNF−group3
T7DNApol
UvdE
HJR−RuvC
NAD−ligase
divDNApol
divDnaB
Polass−SH3
RNaseH1
FLAP
Clamp−RFC
Toprim−DNAG
gp41−DnaB
RecB−like
TopoIV
TopoIV−subA
gp45−clamp
PrimaseZnR
UvsY
FamBDNAPol
gp32−SSB
UvsW−A18
SbcD
SbcC
UvsX−RecA
PolB35exo
gp59−hel−load
gp62−Clloadss
DexA35exo
ATP−DNA−ligase−DBD
DprA
T5orf172−URI
PolI135exo
RuvB−like
minimalDNA_pol13
ATP−DNA−ligase
HJR−EndoVII
EndoV
UvrD−dda
TraR−ZnF
WGR
TFIIE−ZnR
GNAT
HTH
RpoSigma
DsbA
RpoART
T4gp33
RpoSigma−min
rIIB
antisigma
AbrB
SNF−group4
Phd
Rpobetaprime
Rpobetaprime−C
RpobetaC
Rpobeta
Rpogp68
RpobetaN
Rpo−Rad25−like
SNF−group2
Tail−fiber
LaminG
vWA
MoxR
YHYH
HSP20
Hsp10
ClpS−
Clp−Protease
BCS1−AAA
Phautopepchap
PAAR
XkdF−S−Peptidase
Inh
Capsid−protease
DNAJ
HSP70
gp4−hd−comp−REase
Maj−Capsid
Base−wedge
T4gp19−Tailtube
DNA−end−prot
Tail−sheath
Terminase−LS
portal
prohead−protease
Virion−MPTase
PhiKZ−Major−Capsid
Tubulin
Hsp60
AlkB
Alp
ABC−GNAT
MOM
GCHI
QTG
QueE
QueD
QueC
DAM−others
M.MbolIA−rel−DAM
M.MbolIA−like−cpDAM
N4−M.MbolIA−like−DAM
M.EcoKI−DAM−misc
cpM.EcoKI−like−DAM
M.EcoKI−like−DAM
T4−DAM−like
DCMethylase
DAM−minimal
YpsA_SLOG
DHH−cpdease
Nribosyltransf
NAD−synth_kinaseonly
calcineurin−counterint
NADAR−gp30.3
YACar_SLOG
HD−GYP
DUF4326
NAD−synth_Ploop
NADAR−YbiaA
NAPRTase
NAD−synth_NUDIX
cREC
NUDIX−other
MACRO
HD−alarmone_pdease
SIR2
NUDIX−CREca
NADAR
Rnl1−RNA_ligase
Rnl2−RNA_ligase
HAD−PNKa
PNK
calcineurin−repair
CCA−adding
Thgl
PTH2
RlaP
HAD−SAK
KTSC
IF3
RegA
PhoH
2H
PhoH_PINf
PTH1
RtcB
Rnl2−HNH
HD−RNArepair
Rot_TROVE
