# Supplementary material for: Boreal and subarctic freshwaters harbour a diversity of jumbophages
Source: ISME Commun. 2026 Aug 22;6(1):ycag239. doi: 10.1093/ismeco/ycag239 (PMC13573597; doi:10.1093/ismeco/ycag239)
Supplement: Supplementary file 1 [file supplementary_material_ycag239.zip › Supplementary_final.docx]

Supplementary material for:

Boreal and subarctic freshwaters harbour a diversity of jumbophages

Meeri Niemi^1^, Karyna Karneyeva^1^, Lotta-Riina Sundberg^1^, Hanna M. Oksanen^2^, Elina Laanto*^1^

1 Department of Biological and Environmental Science and Nanoscience Center, PO Box 35, FI-40014 University of Jyväskylä, Finland

2 Department of Molecular and Integrative Biosciences, Faculty of Biological and Environmental Sciences, University of Helsinki, Helsinki, Finland.

*corresponding author

Running title: Northern freshwater jumbophages

*Address: Department of Biological and Environmental Science and Nanoscience Center, PO Box 35, FI-40014 University of Jyväskylä, Finland, email: [elina.laanto@jyu.fi](mailto:elina.laanto@jyu.fi)

## Supplementary methods

### Bacterial host 16S taxonomy

For taxonomic identification, the partial 16S rRNA gene was amplified using strain-specific primer pairs (Table S1) and Biotools DNA polymerase (Biotools B&M Labs S.A.). A loopful of bacterial pure culture was suspended in 50 µL dH2O, and 1 µL of the suspension was used as PCR template following an extended initial denaturation step. PCR products were purified (Qiagen PCR Purification Kit) and sequenced with an ABI Prism 3130xl Genetic Analyzer (Applied Biosystems) using BigDye v3.1 (Thermo Fisher Scientific).

Host taxonomy was assigned based on 16S rRNA sequence similarity to reference sequences from the NCBI database. For each host, the three top BLASTn hits were compared; if all corresponded to the same genus, that taxonomy was assigned. To evaluate host relatedness, a maximum likelihood phylogenetic tree was constructed via IQ-TREE using the sequences corresponding to these BLASTn matches. The tree was built applying the best-fit nucleotide substitution model determined by model selection and was supported by 1,000 bootstrap replicates. In the case of host WB09, the top hits included *Duganella zoogloeoides*, *Rugamonas aquatica*, and *Rugamonas rivuli*. Since the latter two species have been proposed for reassignment to the genus *Duganella* [1], the final taxonomy for WB09 was designated as *Duganella*.

### Phage purification and imaging

High-titre phage lysates (10^9^ – 10^10^ PFU mL⁻¹) were precipitated using 10% (w/v) polyethylene glycol (PEG) 6000 (Sigma-Aldrich) and 0.5 M NaCl. After incubating for 60 min with gentle agitation at 4°C, the phage precipitates were centrifuged at 7650 × *g* for 20 min at 4 °C (Fiberlite F21-8 × 50y rotor, Sorvall Lynx 6000 superspeed, Thermo Scientific). Precipitates were dissolved in 20 mM potassium phosphate buffer (pH 7.2) placed on top of a linear 5-20% (w/v) sucrose gradient, and centrifuged for 98 500 × g for 20 min at 15 °C (SW 41 Ti, Optima L-90K ultracentrifuge, Beckman Coulter). The subsequent light-scattering virus band was collected, concentrated (93 800 × g, 2 h, 4 °C, 70.1 Ti rotor, Optima L-90K ultracentrifuge, Beckman Coulter), and resuspended in 20 mM potassium phosphate buffer (pH 7.2). Grids (200 mesh, formvar coated copper grids) were glow-discharged (30 s, 22 mA) to render the surface hydrophilic. For both the virus bands and the concentrated samples, phage preparations were applied to each grid for 1 min. The grid was then stained with 1% (w/v) phosphotungstic acid (pH 7.4) for 2 min. Images were acquired using a JEOL JEM-1400 transmission electron microscope (JEOL Ltd) operated at 80 kV, equipped with a bottom mounted Quemesa CCD camera (4008×2664- pixel resolution). From recorded images, phage particle sizes were measured using ImageJ (version 1.54p) [2].

### Manual phage genome assembly

When automated assembly was unsuccessful, raw reads were processed and assembled manually. Reads were trimmed with Trim Galore (v0.6.10) [3] and quality-checked using FastQC (v0.12.1) [4]. De novo assemblies were generated using either metaviralSPAdes [5] or metaSPAdes [6]. Genome termini were assessed with PhageTerm (v1.0.12) [7]. Reads were remapped to assembled contigs using BWA-MEM2 (v 2.3) [8], and assemblies were polished with Pilon (v1.20) [9]. For jumbophages, Pharokka + Phold annotation was conducted with script retrieved from https://colab.research.google.com/github/gbouras13/phold/blob/main/run_pharokka_and_phold_and_phynteny.ipynb.

### Phylogenetic analysis based on large terminase subunit

A dataset of large terminase subunit (TerL) sequences was compiled from ViPTree output (Accessed July 2025, version 4.0) [10] with dsDNA viruses of prokaryotic hosts chosen as the reference type, yielding a tree containing 827 reference phages showing sequence similarity (SG > 0.02 to either a query genome or to another related reference genome) to new jumbophage sequences. From the initial set, 160 phages were manually selected to represent diverse lineages and host taxa. In addition, TerL sequences from Flavobacteriaceae-infecting phages were retrieved from RefSeq and merged with the new genomes to construct a phylogenetic tree together with the flavophages from our collection. Based on the tree, close relatives of the Flavobacterium jumbophages were selected and added to the final dataset.    Redundancy was reduced using CD-HIT (version 4.8.1) [11] with a 90 % identity threshold, resulting in 101 reference sequences and eight TerL sequences of the newly sequenced jumbophages, yielding a dataset of 109 sequences. Sequences shorter than 70 % of the mean length were excluded to ensure alignment quality, resulting in a final dataset of 105 sequences. Multiple sequence alignment was performed using MAFFT (version 7.526) [12]. The optimal substitution model was selected by IQ-TREE (version 1.6.12) [13] based on the Bayesian Information Criterion (BIC). Phylogenetic reconstruction was conducted with 1,000 bootstrap replicates, and bootstrap > 70 was considered indicative of reliable phylogenetic relationships. Trees were visualised in iTOL v7 (version 7.2.2) [14]. Taxonomic classification data was fetched from ICTV (accessed August 2025), and in the absence of ICTV data, NCBI data was used.

### Detection of nucleus-like structures during infection

Host bacteria were grown to logarithmic phase and infected with phage lysate using a multiplicity of infection (MOI) of 10. After 15, 30, and 60 min, samples (1 mL) were taken, and the cells were pelleted (2350 × g, 10 min), resuspended in 4% (w/v) paraformaldehyde (PFA) in phosphate-buffered saline (PBS), and incubated for 30 min at RT. The fixed cells were washed three times with PBS and stained with 1 μg/mL DAPI (4′,6-diamidino-2-phenylindole, Invitrogen) in 500 μL of PBS for 30 min at room temperature in the dark. After washing with PBS, the cells were resuspended in 100 μL of PBS. For imaging, 2 μL of sample was added onto a 1% (w/v) low-melting agarose pad placed on a glass microscope slide and covered with a coverslip. Images were acquired on a Nikon A1R confocal laser-scanning microscope together with the NIS-Elements confocal imaging software, using a CFI Plan Apo VC 60× water-immersion objective with 4× averaging. Uninfected controls were processed in parallel. Phage nucleus formation was inferred from the appearance of centrally located DAPI-stained structures.

### Comparative Genomic Analysis

Whole-genome BLASTn [15] searches against the INPHARED database (April 2025 release) [16] identified the closest genomes, which were selected as references for downstream comparative analyses. Genome FASTA sequences were submitted to ViPTree server (accessed October 2025) [10] to generate tBLASTx-based proteomic trees, which were visualised in R using ggtree (v3.16.3) [17] and ggplot2 (v3.5.2) [18]. Intergenomic similarities for closest phages were calculated using VIRIDIC (accessed September 2025) [19]. VIRIDIC was also used to calculate genomic similarity between jumbophages. Further, VirClust server (accessed October 2025) [20] was used for protein-cluster-based phylogenetic analysis of Flavobacterium-infecting phages in the collection with recommended criteria [21].

## Supplementary Results

### Functional annotation profiles of the phage genomes

Phages were annotated through the Phagenomics workflow, which uses several reference databases: Prokaryotic Virus Remote Homologous Groups (PHROGs), Clusters of Orthologous Genes (COGs), prokaryotic Virus Orthologous Groups (pVOGs), and Phage Artificial Neural Networks (PHANNs). For each phage genome, the proportion of genes assigned to each database was calculated to assess annotation coverage (Fig. S8). Annotation coverage varied widely across the collection. Overall, the fraction of unannotated genes increased with genome size, whereas the proportion of PHROGs hits declined. Larger phages more frequently contained genes related to DNA, RNA, and nucleotide metabolism, as well as those grouped within the broad “other” PHROGs category.

Among the largest genomes, there was also a slight increase of genes classified as morons, auxiliary metabolic genes (AMGs), and host-takeover functions. The *Pseudomonas* jumbophage Ahti showed a notably higher number of “head and packaging” genes compared to other phages. The largest jumbophages (Aino, FKo-1, and FTs-1) displayed a greater proportion of COG-level matches, including the ribosomal protein L7/L12 in Aino. The detailed distribution of annotation categories across the different databases is provided in Supplementary Figure S8.

### Intergenomic similarity among jumbophages

Pairwise genome comparisons using VIRIDIC showed that the jumbophages in our collection are largely distinct from one another, exhibiting low intergenomic similarity across most pairs (Fig. S7). The highest relatedness occurred among the Flavobacterium-infecting phages. FKo-2 and FKy-2 shared an intergenomic similarity of 80.9 %, while FKj-2 showed moderate similarity to both FKo-2 and FKy-2 (48.7 % each). A more distant relationship was observed between FKo-1 and FTs-1 (27.0 %). All remaining pairwise comparisons yielded values below 10 %, indicating substantial genomic divergence. These results highlight the diversity of the isolated jumbophages, with only a subset clustering into groups.

### tRNA counts

The number of predicted tRNA genes varied substantially among genomes, ranging from 0 to 62 (median = 0; mean = 7.05; n = 40) (Fig. S9). Across the collection, tRNA count correlated positively with genome length (Pearson r = 0.715, 95 % CI = 0.572–0.863, p = 2.14 × 10⁻⁷; Spearman ρ = 0.61, p < 0.0001). However, this relationship weakened among the largest genomes: five of the eight jumbophages encoded few or no tRNAs.

### Unknown genes

Since phages larger than 200 kb were initially annotated with an average of approximately 66 % unknown genes, we tested an alternative pipeline that combined Pharokka with Phold to improve functional annotation of the jumbophages. Phold supplements sequence-based annotation with protein structural homology searches, enabling functional inference even for highly divergent viral proteins. Most of the genes identified by Phold were assigned to structural and metabolic or enzymatic categories (Table S4). Overall, Phold consistently expanded the functional annotation of jumbophage genomes.

## Supplementary figures

Supplementary Figure S1. Phylogenetic tree based on BLASTn hits for partial 16S rRNA gene sequences of bacterial hosts isolated in this work. Species within the same genus are marked in different colours (see colour chart).

Alt text: Phylogenetic tree of bacterial host 16S rRNA gene sequences, showing host isolates grouped mainly by bacterial genus.

Supplementary Figure S2. Hierarchical tree and heatmap representation of protein cluster (PC) distribution in *Flavobacterium* phage genomes, generated using the VirClust server. Rows correspond to individual viral genomes, and columns to individual PCs. PC IDs are indicated at the bottom of the heatmap. Colors indicate the number of occurrences of each PC per genome, with white representing absence (see legend). To the right of the heatmap, genome-specific statistics are shown: genome length and the proportion of PCs shared with other genomes (dark grey) relative to the total number of PCs in the genome (light grey bar).

Alt text: Protein-cluster heatmap of Flavobacterium phages, showing two main jumbophage groups with shared protein-cluster patterns.

Supplementary Figure S3. Pairwise intergenomic identities among freshwater jumbophage genomes, calculated with VIRIDIC. Values represent percentage similarity, with colour shading indicating similarity level (0–100 %). Phage names are colour-coded by isolation host genus.

Alt text: VIRIDIC heatmap showing pairwise genome similarity among freshwater jumbophages, with high similarity only within two Flavobacterium phage groups.

Supplementary Figure S4. Transmission electron microscopy of negatively stained jumbophages. A) *Flavobacterium* phage FTs-1 B) *Flavobacterium* phage FKj-2
C) *Flavobacterium* phage FKy-2. All stained using phosphotungstic acid. Scale bars: 100 nm.

Alt text: Transmission electron micrographs of additional Flavobacterium jumbophages, showing large myovirus-like particles with capsids and tails.

Supplementary Figure S5. Transmission electron microscopy of negatively stained phages (A) Tursas (B) Ilmatar, (C) Mielikki, and (E) Kaleva. (D) Kaleva virions attached to its host *Exiguobacterium* sp. 5 min post-infection, fixed with 3 % glutaraldehyde. All stained using phosphotungstic acid. Scale bars: 100 nm.

Alt text: Transmission electron micrographs of smaller freshwater phages, showing varied particle morphologies and phage particles attached to a bacterial host cell.

Supplementary Figure S6. Efficiency of plating of jumbophages on jumbophage host bacteria assessed by spot assay. Phylogenetic tree based on partial 16S rRNA gene sequences of bacterial hosts. Pink shading marks the original isolation host for each phage.

^1^ Values indicate the average phage titre (PFU mL⁻¹) on each tested host (n=3).

^2^ Plus sign (+), infection without countable plaques

^3^ Minus sign (–), no detectable infection.

Alt text: Host-range table and bacterial phylogeny showing that jumbophages infect only hosts from the same bacterial genus as their isolation host.

Supplementary Figure S7. Comparison of plaque morphologies of the jumbophages on their isolation host bacteria (scale bars: 5 mm) and the plaques of Flavobacterium phage FLe-1 (genome size 81547 bp) on its host *Flavobacterium* sp. B176.

Alt text: Plaque image of a smaller Flavobacterium phage, showing larger and more visible plaques than the jumbophage plaques.

Supplementary Figure S8. Maximum likelihood phylogenetic tree of major capsid protein sequences of freshwater jumbophages (stars) and 82 representative reference phages spanning diverse viral lineages. Jumbophages are highlighted in pink. Phylogenetic inference was performed using IQ-TREE with the best-fit evolutionary model selected by model testing and 1,000 ultrafast bootstrap replicates. The tree is midpoint-rooted and visualised in iTOL v7. The outer ring is colour-coded by phage family or genus (when not assigned to a family).

Alt text: Major capsid protein phylogenetic tree showing freshwater jumbophages distributed across multiple branches among reference phages.

Supplementary Figure S9. Functional annotation profiles of the isolated phage genomes. Stacked bar plots showing the proportions of predicted protein functions assigned by different annotation databases. Colours correspond to databases (legend, right). Phages are ordered by increasing genome size; the middle bar plot displays genome lengths, colour-coded by the host genus.

Alt text: Stacked bar plots showing functional annotation categories across phage genomes, with larger genomes containing high proportions of unknown genes.

Supplementary Figure S10. Relationship between phage genome (n=40) length and tRNA count. Each point represents a phage genome, coloured by the phage host genus.

Alt text: Scatter plot showing a positive correlation between phage genome length and number of predicted tRNA genes.

Supplementary Figure S11. Presence of proposed core jumbophage gene products in freshwater jumbophages*.* Heatmaps show the presence (pink) or absence (white) of hallmark jumbophage gene products [22]. Blue outlines mark discrepancies between the two annotation pipelines. A) Phagenomics annotation. B) Pharokka + Phold annotation.

Alt text: Heatmaps showing presence and absence of broadly conserved jumbophage protein families across the freshwater jumbophage genomes.

Supplementary Figure S12. Confocal microscopy of cells infected with the eight jumbophages at 60 min post-infection, alongside the corresponding uninfected control cells. Of the eight jumbophages, only Ahti shows distinct localised DAPI staining, whereas the other infections show more diffuse DNA staining.

Alt text: Confocal microscopy images of jumbophage-infected cells, showing distinct localised DAPI staining only in Ahti-infected cells.

Supplementary Figure S13. Comparative analysis of core gene product homologues between phage Ahti and representative nucleus-forming phages. Heatmaps show BLASTp (E-value threshold 0.001) bit scores between the predicted proteins of *Pseudomonas-*infecting phage Ahti and a database of 21 core gene products characteristic of nucleus-forming phages [23] from three reference genomes: ΦKZ (left), Goslar (centre), and RAY (right). Gene products are colour-coded by predicted functional class, and text labels indicate putative functions of the corresponding reference genes where specific functional annotations are available. Unlabelled grey entries correspond to core genes annotated as hypothetical proteins [23].

Alt text: Heatmaps comparing Ahti proteins to nucleus-forming phage core proteins from ΦKZ, Goslar, and RAY, with strongest matches to ΦKZ.

## Supplementary Tables

Supplementary Table S1. Detailed list of phage-host systems isolated and used in the study (separate excel file)

Supplementary Table S2. Average titres for each jumbophage lysate in this study, achieved following propagation on the respective isolation hosts.

| Phage | Host | Average titre | n ^1^ |
| --- | --- | --- | --- |
| FKo-1 | *Flavobacterium* sp. B28 | 2.84 × 10⁸ | 4 |
| FTs-1 | *Flavobacterium* sp. B121 | 1.50 × 10¹⁰ | 5 |
| FKj-2 | *Flavobacterium* sp. B130 | 4.68 × 10¹⁰ | 5 |
| Lampi | *Janthinobacterium* sp. B140 | 4.54 × 10¹⁰ | 5 |
| Aino | *Herbaspirillum* sp. B21 | 3.34 × 10¹¹ | 3 |
| FKo-2 | *Flavobacterium* sp. B28 | 5.00 × 10⁹ | 3 |
| FKy-2 | *Flavobacterium* sp. B223 | 3.30 × 10¹⁰ | 7 |
| Ahti | *Pseudomonas* sp. WB02 | 8.53 × 10¹⁰ | 6 |

^1^ measurements are averaged from *n* lysates

Supplementary Table S3. Phagenomics annotations. (separate excel file)

Supplementary Table S4. Pharokka + Phold annotations of jumbophages. (separate excel file)

Supplementary Table S5. List of reference phages used in building a large terminase subunit based phylogenetic tree, and their accession numbers. (separate excel file)

Supplementary Table S6. Jumbophage capsid diameters (facet-to-facet) and tail lengths measured from negatively stained particles imaged with TEM.

| Phage | Capsid  (facet-to-facet) (nm) | Tail (nm) | *n* ^1^ |
| --- | --- | --- | --- |
| Aino | 115 | 112 | 1 |
| FKo-1 | 134 | 162 | 3 |
| FTs-1 | 132 | 146 | 1 |
| FKj-2 | 140 | 170 | 2 |
| Lampi | 146 | 141 | 3 |
| FKy-2 | 121 | 170 | 2 |
| Ahti | 129 | 187 | 3 |

^1^ measurements are averaged from *n* individual virions

Supplementary Table S7. KEGG metabolic categories of respective AMGs in eight jumbophages predicted by VIBRANT.

| **Aino** |  |  |
| --- | --- | --- |
| **AMG count** | **AMG KEGG orthology** | **AMG KEGG orthology name** |
| **1** | K00859 | coaE; dephospho-CoA kinase [EC:2.7.1.24] |
| **1** | K03462 | NAMPT; nicotinamide phosphoribosyltransferase [EC:2.4.2.12] |
| **1** | K00858 | ppnK, NADK; NAD+ kinase [EC:2.7.1.23] |
| **1** | K00954 | E2.7.7.3A, coaD, kdtB; pantetheine-phosphate adenylyltransferase [EC:2.7.7.3] |
| **1** | K10026 | queE; 7-carboxy-7-deazaguanine synthase [EC:4.3.99.3] |
| **1** | K01916 | nadE; NAD+ synthase [EC:6.3.1.5] |
| **1** | K01779 | racD; aspartate racemase [EC:5.1.1.13] |
| **1** | K01737 | queD, ptpS, PTS; 6-pyruvoyltetrahydropterin/6-carboxytetrahydropterin synthase [EC:4.2.3.12 4.1.2.50] |
| **1** | K00287 | DHFR, folA; dihydrofolate reductase [EC:1.5.1.3] |
| **1** | K09007 | folE2; GTP cyclohydrolase IB [EC:3.5.4.16] |
| **1** | K13522 | K13522, nadM; bifunctional NMN adenylyltransferase/nudix hydrolase [EC:2.7.7.1 3.6.1.-] |
| **FTs-1** |  |  |
| **AMG count** | **AMG KEGG orthology** | **AMG KEGG orthology name** |
| **1** | K00472 | P4HA; prolyl 4-hydroxylase [EC:1.14.11.2] |
| **1** | K00287 | DHFR, folA; dihydrofolate reductase [EC:1.5.1.3] |
| **1** | K13522 | K13522, nadM; bifunctional NMN adenylyltransferase/nudix hydrolase [EC:2.7.7.1 3.6.1.-] |
| **1** | K01939 | purA, ADSS; adenylosuccinate synthase [EC:6.3.4.4] |
| **1** | K03462 | NAMPT; nicotinamide phosphoribosyltransferase [EC:2.4.2.12] |
| **1** | K00860 | cysC; adenylylsulfate kinase [EC:2.7.1.25] |
| **1** | K02377 | TSTA3, fcl; GDP-L-fucose synthase [EC:1.1.1.271] |
| **1** | K01711 | gmd, GMDS; GDPmannose 4,6-dehydratase [EC:4.2.1.47] |
| **FKo-1** |  |  |
| **AMG count** | **AMG KEGG orthology** | **AMG KEGG orthology name** |
| **1** | K01885 | EARS, gltX; glutamyl-tRNA synthetase [EC:6.1.1.17] |
| **1** | K00287 | DHFR, folA; dihydrofolate reductase [EC:1.5.1.3] |
| **1** | K03462 | NAMPT; nicotinamide phosphoribosyltransferase [EC:2.4.2.12] |
| **1** | K01791 | wecB; UDP-N-acetylglucosamine 2-epimerase (non-hydrolysing) [EC:5.1.3.14] |
| **1** | K00859 | coaE; dephospho-CoA kinase [EC:2.7.1.24] |
| **1** | K13522 | K13522, nadM; bifunctional NMN adenylyltransferase/nudix hydrolase [EC:2.7.7.1 3.6.1.-] |
| **1** | K01939 | purA, ADSS; adenylosuccinate synthase [EC:6.3.4.4] |
| **Ahti** |  |  |
| **AMG count** | **AMG KEGG orthology** | **AMG KEGG orthology name** |
| **1** | K06920 | queC; 7-cyano-7-deazaguanine synthase [EC:6.3.4.20] |
| **1** | K00287 | DHFR, folA; dihydrofolate reductase [EC:1.5.1.3] |
| **1** | K03462 | NAMPT; nicotinamide phosphoribosyltransferase [EC:2.4.2.12] |
| **FKo-2** |  |  |
| **AMG count** | **AMG KEGG orthology** | **AMG KEGG orthology name** |
| **1** | K00287 | DHFR, folA; dihydrofolate reductase [EC:1.5.1.3] |
| **2** | K02078 | acpP; acyl carrier protein |
| **FKj-2** |  |  |
| **AMG count** | **AMG KEGG orthology** | **AMG KEGG orthology name** |
| **1** | K00287 | DHFR, folA; dihydrofolate reductase [EC:1.5.1.3] |
| **1** | K02078 | acpP; acyl carrier protein |
| **FKy-2** |  |  |
| **AMG count** | **AMG KEGG orthology** | **AMG KEGG orthology name** |
| **1** | K00287 | DHFR, folA; dihydrofolate reductase [EC:1.5.1.3] |
| **1** | K02078 | acpP; acyl carrier protein |
| **Lampi** |  |  |
| **AMG count** | **AMG KEGG orthology** | **AMG KEGG orthology name** |
| **1** | K00287 | DHFR, folA; dihydrofolate reductase [EC:1.5.1.3] |

Supplementary Table S8. Gene list with putative role in representatives of detected AMG islands in *Flavobacterium* jumbophages, FTs-1 for NAD and FKo-2 for host-interface.

| Phage | GeneID | Annotation | Putative role in AMG island |
| --- | --- | --- | --- |
| FTs-1 | 224 | hypothetical protein |  |
|  | 223 | hypothetical protein |  |
|  | 222 | Predicted membrane chloride channel, bestrophin family | Ion channel |
|  | 221 | hypothetical protein |  |
|  | 220 | hypothetical protein |  |
|  | 219 | dihydrofolate reductase | The sequence clusters with annotated dihydrofolate reductase–like proteins (HHpred), consistent with a role in folate/NAD redox homeostasis |
|  | 218 | hypothetical protein |  |
|  | 217 | tRNA amidotransferase | translation modulation |
|  | 216 | phosphoesterase | Could degrade NAD, NMN or ppGpp-like signals. The protein contains the hallmark HD catalytic dyad characteristic of HD-domain phosphohydrolases. However, it lacks the canonical HD-xx-H motif found in some HD-superfamily subclasses. |
|  | 215 | AAA ATPase domain-containing protein | Remodeling, activating or regulating NAD metabolism during infection |
|  | 214 | NMNAT-like nucleotidyltransferase (Citidyltransferase) | Key NAD Salvage AMG, Converts NMN → NAD intermediate, direct partner to NAMPT |
|  | 213 | ATPase | Energy provision/remodelling and trafficking/regulation |
|  | 212 | DNA primase/helicase | Together with ATPase and AAA ATPase could be associated with replication energy demand, possibly nucleotidyl transfer reaction coupling |
|  | 211 | hypothetical protein |  |
|  | 210 | nicotinamide phosphoribosyl transferase | Central to NAD salvage pathway |
|  |  |  |  |
| FKo-2 | 248 | hypothetical protein |  |
|  | 249 | hypothetical protein |  |
|  | 250 | NAD-dependent DNA ligase | phage-encoded NAD-dependent ligases can support genome replication/repair |
|  | 251 | hypothetical protein |  |
|  | 252 | hypothetical protein |  |
|  | 253 | hypothetical protein |  |
|  | 254 | DNA helicase | assist replication under host stress, interact with host replication/transcription machinery |
|  | 255 | hypothetical protein |  |
|  | 256 | hypothetical protein |  |
|  | 257 | hypothetical protein |  |
|  | 258 | metal-dependent phosphohydrolase | ppGpp, enzymatic hydrolase, likely metal-dependent; typical functions include cleavage of phosphoanhydride/ester bonds, possibly: periplasmic nucleotide turnover or modulation of signaling phosphates |
|  | 259 | hypothetical protein |  |
|  | 260 | hypothetical protein |  |
|  | 261 | Acyl carrier –like protein | ACPs suggest roles in lipid modifications or acylation reactions |
|  | 262 | acyl carrier protein | ACPs suggest roles in lipid modifications or acylation reactions |
|  | 263 | hypothetical protein |  |
|  | 264 | Lipoprotein candidate (hypothetical protein) | strong candidate surface/extracellular effector; possibly binds polysaccharides |
|  | 265 | Putative secreted protein (hypothetical protein) | likely lipoprotein anchored to membrane; possible adhesin or polysaccharide-degrading enzyme |
|  | 266 | Hypothetical protein (77ORF036 CDS) |  |

Supplementary Table S9. Putative anti-defence systems identified in the phage genomes using DefenseFinder v2.0.2. Predictions were performed using protein annotations generated with Phagenomics and, for jumbophages, additionally with Pharokka + Phold. Each row represents an individual DefenseFinder profile hit associated with a predicted anti-defence system. The table lists the phage, annotation source, predicted anti-defence type and subtype, matched DefenseFinder profile, protein ID, hit position, and HMM profile-match statistics, including E-value, score, profile coverage, and sequence coverage. Profile coverage indicates the fraction of the HMM profile covered by the hit, while sequence coverage indicates the fraction of the protein sequence covered by the profile match. For systems defined by multiple profiles, separate rows correspond to the individual component profiles of the same predicted system.

(separate excel file)

Supplementary Table S10. Amino acid identity between Ahti proteins and nucleus-forming phage core gene products. BLASTp hits between the Ahti proteome and core gene products from ΦKZ, Goslar, and RAY are shown. Core gene numbers and putative functions are based on Prichard et al. (2023). For each hit, the table lists the corresponding Ahti protein, reference phage protein, amino acid identity, alignment length, query and reference alignment ranges, E-value, and bit score.

(separate excel file)

Supplementary Table S11. BLASTp search for nucleus-forming phage marker proteins in Ahti. BLASTp searches were performed using PicA/Imp1 protein import markers and RNA polymerase-associated marker proteins from nucleus-forming reference phages ΦKZ, Goslar, and RAY. The table lists the best Ahti hit for each reference marker, including amino acid identity, alignment length, query and reference coverage, E-value, and bit score. Hits are interpreted as candidate homologues based on sequence similarity and should not be considered experimental confirmation of function.

(separate excel file)

Supplementary Table S12. Whole-proteome BLASTp comparison of Ahti against nucleus-forming reference phages. Whole-proteome BLASTp searches were performed using the Ahti proteome as query and the proteomes of ΦKZ, Goslar, and RAY as reference databases. For each reference phage, the table summarises the number and proportion of Ahti proteins with BLASTp hits, median amino acid identity, median query and reference coverage, median bit score, total bit score, and the number of Ahti proteins for which that reference phage represented the best match among the three tested references. Only hits meeting the summary filter used in the analysis were included in hit-count and median summaries (E-value ≤ 1×10⁻³ and alignment length ≥30 aa). The comparison was used to assess the protein-level relatedness of Ahti to selected nucleus-forming phages. Metrics are based on sequence similarity and do not imply species-level relatedness.

| Reference phage | Ahti proteins with BLASTp hits (n) | Ahti proteins with BLASTp hits (%) | Median amino acid identity (%) | Median Ahti protein coverage (%) | Median reference protein coverage (%) | Median bit score | Total bit score | Ahti proteins for which this reference was the best match |
| --- | --- | --- | --- | --- | --- | --- | --- | --- |
| ΦKZ | 175 | 37.55 | 40.42 | 95.65 | 95.15 | 150.0 | 46981.7 | 173 |
| RAY | 81 | 17.38 | 29.41 | 88.08 | 86.23 | 115.0 | 13277.3 | 8 |
| Goslar | 75 | 16.09 | 30.21 | 86.54 | 82.48 | 120.0 | 12063.6 | 4 |

## References

1.	Jeon D, Kim IS, Choe H *et al.* Duganella aceris sp. nov., isolated from tree sap and proposal to transfer of Rugamonas aquatica and Rugamonas rivuli to the genus Duganella as Duganella aquatica comb. nov., with the emended description of the genus Rugamonas. *Arch Microbiol* 2021;**203**(6):2843–52. https://doi.org/10.1007/s00203-021-02191-z.

2.	Schindelin J, Arganda-Carreras I, Frise E *et al.* Fiji: an open-source platform for biological-image analysis. *Nat Methods* 2012;**9**(7):676–82. https://doi.org/10.1038/nmeth.2019.

3.	Krueger F, James F, Ewels P *et al.* *FelixKrueger/TrimGalore: V0.6.10 - Add Default Decompression Path*. published online 2 Feb. 2023. https://doi.org/10.5281/zenodo.7598955.

4.	Andrews S. Babraham Bioinformatics - FastQC A Quality Control tool for High Throughput Sequence Data. 2010. https://www.bioinformatics.babraham.ac.uk/projects/fastqc/ (13 Oct. 2025, date last accessed).

5.	Antipov D, Raiko M, Lapidus A *et al.* MetaviralSPAdes: assembly of viruses from metagenomic data. *Bioinformatics* 2020;**36**(14):4126–9. https://doi.org/10.1093/bioinformatics/btaa490.

6.	Nurk S, Meleshko D, Korobeynikov A *et al.* metaSPAdes: a new versatile metagenomic assembler. *Genome Res* 2017;**27**(5):824–34. https://doi.org/10.1101/gr.213959.116.

7.	Garneau JR, Depardieu F, Fortier LC *et al.* PhageTerm: a tool for fast and accurate determination of phage termini and packaging mechanism using next-generation sequencing data. *Sci Rep* 2017;**7**(1):8292. https://doi.org/10.1038/s41598-017-07910-5.

8.	Vasimuddin Md, Misra S, Li H *et al.* Efficient Architecture-Aware Acceleration of BWA-MEM for Multicore Systems. *2019 IEEE International Parallel and Distributed Processing Symposium (IPDPS)* May 2019:314–24. https://doi.org/10.1109/IPDPS.2019.00041.

9.	Walker BJ, Abeel T, Shea T *et al.* Pilon: An Integrated Tool for Comprehensive Microbial Variant Detection and Genome Assembly Improvement. *PLOS ONE* 2014;**9**(11):e112963. https://doi.org/10.1371/journal.pone.0112963.

10.	Nishimura Y, Yoshida T, Kuronishi M *et al.* ViPTree: the viral proteomic tree server. *Bioinformatics* 2017;**33**(15):2379–80. https://doi.org/10.1093/bioinformatics/btx157.

11.	Fu L, Niu B, Zhu Z *et al.* CD-HIT: accelerated for clustering the next-generation sequencing data. *Bioinformatics* 2012;**28**(23):3150–2. https://doi.org/10.1093/bioinformatics/bts565.

12.	Katoh K, Standley DM. MAFFT Multiple Sequence Alignment Software Version 7: Improvements in Performance and Usability. *Mol Biol Evol* 2013;**30**(4):772–80. https://doi.org/10.1093/molbev/mst010.

13.	Minh BQ, Schmidt HA, Chernomor O *et al.* IQ-TREE 2: New Models and Efficient Methods for Phylogenetic Inference in the Genomic Era. *Mol Biol Evol* 2020;**37**(5):1530–4. https://doi.org/10.1093/molbev/msaa015.

14.	Letunic I, Bork P. Interactive Tree of Life (iTOL) v6: recent updates to the phylogenetic tree display and annotation tool. *Nucleic Acids Res* 2024;**52**(W1):W78–82. https://doi.org/10.1093/nar/gkae268.

15.	Camacho C, Coulouris G, Avagyan V *et al.* BLAST+: architecture and applications. *BMC Bioinformatics* 2009;**10**(1):421. https://doi.org/10.1186/1471-2105-10-421.

16.	Cook R, Brown N, Redgwell T *et al.* INfrastructure for a PHAge REference Database: Identification of Large-Scale Biases in the Current Collection of Cultured Phage Genomes. *Phage (New Rochelle)* 2021;**2**(4):214–23. https://doi.org/10.1089/phage.2021.0007.

17.	Yu G. Using ggtree to Visualize Data on Tree-Like Structures. *Curr Protoc Bioinformatics* 2020;**69**(1):e96. https://doi.org/10.1002/cpbi.96.

18.	Wickham H. *Ggplot2: Elegant Graphics for Data Analysis*. 2016. https://ggplot2.tidyverse.org.

19.	Moraru C, Varsani A, Kropinski AM. VIRIDIC-A Novel Tool to Calculate the Intergenomic Similarities of Prokaryote-Infecting Viruses. *Viruses* 2020;**12**(11):1268. https://doi.org/10.3390/v12111268.

20.	Moraru C. VirClust-A Tool for Hierarchical Clustering, Core Protein Detection and Annotation of (Prokaryotic) Viruses. *Viruses* 2023;**15**(4):1007. https://doi.org/10.3390/v15041007.

21.	Turner D, Kropinski AM, Adriaenssens EM. A Roadmap for Genome-Based Phage Taxonomy. *Viruses* 2021;**13**(3):506. <https://doi.org/10.3390/v13030506>.

22. Iyer LM, Anantharaman V, Krishnan A, Burroughs AM, Aravind L. Jumbo Phages: A Comparative Genomic Overview of Core Functions and Adaptions for Biological Conflicts. Viruses 2021;13(1):63. <https://doi.org/10.3390/v13010063>.

23. Prichard A, Lee J, Laughlin TG, Lee A, Thomas KP, Sy AE et al. Identifying the core genome of the nucleus-forming bacteriophage family and characterization of Erwinia phage RAY. Cell Reports 2023;42(5). https://doi.org/10.1016/j.celrep.2023.112432.
